# Obeticholic acid alleviates lupus pathology by inhibiting T follicular helper cell differentiation

**DOI:** 10.1038/s44321-026-00478-6

**Published:** 2026-07-03

**Authors:** Minjung Kang, Jiho Park, Jongeun Lee, Sung Won Kim, Yoontae Lee

**Affiliations:** 1 Department of Life Sciences, Pohang University of Science and Technology (POSTECH), Pohang, Republic of Korea; 2 Department of Otolaryngology-Head and Neck Surgery, Seoul St. Mary’s Hospital, College of Medicine, The Catholic University of Korea, Seoul, Republic of Korea

**Keywords:** Immunology

## Abstract

Systemic lupus erythematosus (SLE) is a chronic autoimmune disease characterized by aberrant germinal center (GC) reactions and autoantibody production. Expansion of T follicular helper (T_FH_) cells is a hallmark of SLE that contributes to disease progression. Accordingly, T_FH_ cells represent a promising therapeutic target for SLE. Here, we repurposed obeticholic acid (OCA), an FDA-approved drug for primary biliary cholangitis, as a potential treatment for SLE. OCA selectively inhibited the differentiation of T_FH_ cells both in vitro and in vivo by suppressing the transcription factor ETV5, thereby downregulating *SPP1*, a key ETV5 target that promotes the development of T_FH_ cells. In lupus-prone mice, OCA treatment reduced T_FH_- and GC B-cell populations and alleviated lupus-like manifestations, including autoantibody production and tissue pathology. These findings highlight OCA as a promising immunomodulatory candidate for SLE, providing avenues for devising a therapeutic strategy targeting the T_FH_ cell–GC axis in systemic autoimmunity.

## Synopsis

Obeticholic acid suppresses T follicular helper cell differentiation in murine and human CD4^+ ^T cells by inhibiting the ETV5-SPP1 axis and alleviates lupus pathology across multiple mouse models, supporting its repurposing for T follicular helper cell-driven systemic autoimmunity.

- Obeticholic acid selectively suppresses T follicular helper cell differentiation in murine and human CD4^+ ^T cells.
- Obeticholic acid suppresses SPP1/OPN expression by inhibiting ETV5 binding to the *Spp1* promoter without altering ETV5 expression.
- Genetic and rescue experiments establish the ETV5-SPP1 axis as a key mediator of obeticholic acid-dependent suppression of T follicular helper cell differentiation.
- Obeticholic acid alleviates lupus-like pathology across multiple mouse models, accompanied by reduced germinal center B cell responses, autoantibody production, and tissue pathology.

## Abstract (web summary)

Obeticholic acid suppresses T follicular helper cell differentiation in murine and human CD4^+ ^T cells by inhibiting the ETV5-SPP1 axis and alleviates lupus pathology across multiple mouse models, supporting its repurposing for T follicular helper cell-driven systemic autoimmunity.

## Graphical abstract

**Figure:**
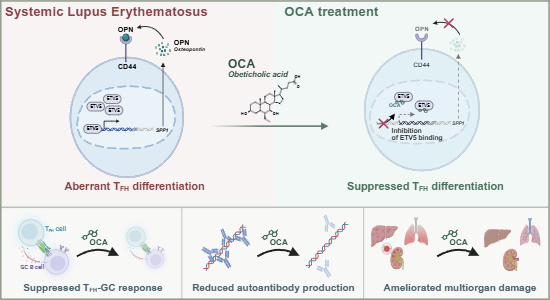

**Figure:**
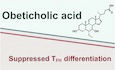

The paper explained **Problem**

Systemic lupus erythematosus (SLE) is a chronic autoimmune disease in which the immune system loses tolerance to self and produces autoantibodies, particularly against nuclear antigens. A major driver of disease is the abnormal expansion of T follicular helper (T_FH_) cells, which promote germinal center (GC) responses, autoreactive B cell activation, and pathogenic autoantibody production. Current therapies for SLE are largely nonspecific and often rely on broad immunosuppression, highlighting the need for more selective approaches that target key pathogenic immune pathways.

**Results**

This study identifies obeticholic acid (OCA), an FDA-approved drug currently used for primary biliary cholangitis, as a potential therapeutic agent for lupus by showing that it selectively restrains T_FH_ cell differentiation. In both murine and human T cells, OCA suppressed the T_FH_ cell program through suppression of the ETV5–SPP1 pathway. Mechanistically, OCA impaired ETV5 binding to the *Spp1* promoter without changing ETV5 abundance, leading to reduced expression of osteopontin, a key mediator of T_FH_ cell differentiation. Genetic and rescue experiments further supported a key role for the ETV5–SPP1 axis in mediating this inhibitory effect. Consistent with these mechanistic findings, OCA treatment attenuated T_FH_ and GC B cell responses, reduced autoantibody production, and improved tissue pathology across multiple mouse models of lupus.

**Impact**

These findings support the repurposing potential of OCA in SLE and identify the ETV5–SPP1 axis as a therapeutically relevant pathway in T_FH_ cell-driven autoimmunity. By restraining a central pathogenic immune circuit, OCA may offer a more targeted therapeutic strategy than conventional broad immunosuppression. More broadly, this study provides a rationale for exploring ETV5-directed therapeutic strategies in lupus and related autoimmune diseases.

## Introduction

Systemic lupus erythematosus (SLE) is a chronic, multisystem autoimmune disease characterized by the loss of immunological self-tolerance and production of autoantibodies, particularly against nuclear antigens. These autoantibodies form immune complexes that deposit in tissues and trigger complement activation, leading to inflammation and organ damage, most notably in the kidneys (lupus nephritis), skin, and joints (Kaul et al, 2016; Tsokos, 2011). At the cellular level, aberrant germinal center (GC) activity plays a central role in SLE pathogenesis by promoting the survival and expansion of autoreactive B cells, which eventually mature into long-lived plasma cells that sustain autoantibody production (Vinuesa et al, 2009).

T follicular helper (T_FH_) cells are critical for initiating and sustaining GC reactions. These cells are characterized by the BCL6, CXCR5, ICOS, and PD-1 expression and IL-21 secretion. CXCR5 directs the migration of T_FH_ cells into B-cell follicles, where they provide critical help to GC B cells (Choi et al, 2024; Crotty, 2014). In lupus-prone mouse models and in patients with SLE, the T_FH_ cell population is expanded and correlates with increased GC B cell responses, autoantibody titers, and disease severity (Craft, 2012; Morita et al, 2011; Simpson et al, 2010). These observations suggest that the T_FH_–GC axis is a key driver of SLE and can be a promising target for therapeutic intervention.

Current therapies for SLE are largely nonspecific. Although immunosuppressive agents, such as glucocorticoids, antimalarials, and B cell-depleting monoclonal antibodies (e.g., belimumab), can reduce disease flares and antibody titers, they cause general impairment of immune function, leading to increased infection risk and incomplete disease control (Fanouriakis et al, 2024; Morand and Jones, 2026). Moreover, conventional therapies do not directly address the problem posed by aberrant T cell help that sustains pathological GC activity. These limitations underscore the need for therapeutic strategies that can selectively modulate the differentiation or function of T_FH_ cells.

ETS variant 5 (ETV5) was recently identified as a key regulator of T_FH_ cell differentiation (Park et al, 2024; Park et al, 2017). Its expression in T_FH_ cells is higher than in non-T_FH_ cells, and it promotes the expression of T_FH_-associated genes, including *Spp1*, which encodes osteopontin (OPN)—a secreted glycoprotein that binds to CD44 to promote the differentiation of T_FH_ cells (Park et al, 2024). Genetic deletion of *Etv5* in T cells leads to a significant reduction in T_FH_ cell abundance, impaired GC formation, and attenuated autoimmunity in murine models, highlighting ETV5 as a potential upstream regulator of the T_FH_ program (Park et al, 2024). Despite its importance, no clinically available inhibitors of ETV5 exist, leaving the therapeutic potential of this target untapped.

Obeticholic acid (OCA) is a clinically approved farnesoid X receptor (FXR) agonist used to treat primary biliary cholangitis (PBC) (Ali and Lindor, 2016). Beyond its metabolic and hepatic roles, OCA exhibits anti-inflammatory and tumor-suppressive properties in various disease contexts (Attia et al, 2017; Gadaleta et al, 2011; Li et al, 2020). Notably, in a recent study using prostate cancer models, we showed that OCA suppresses ETV5 activity by interfering with its DNA binding, leading to the downregulation of ETV5 target genes and attenuation of cancer progression (Lee et al, 2025). Based on these findings, we hypothesized that OCA could inhibit ETV5 in T cells and thereby regulate T_FH_-mediated autoimmune responses. In this study, we aimed to test this hypothesis by investigating the effects of OCA on T_FH_ cell responses and lupus pathogenesis in murine models.

## Results

### OCA restrains T_FH_ cell differentiation via ETV5

**Figure 1:**
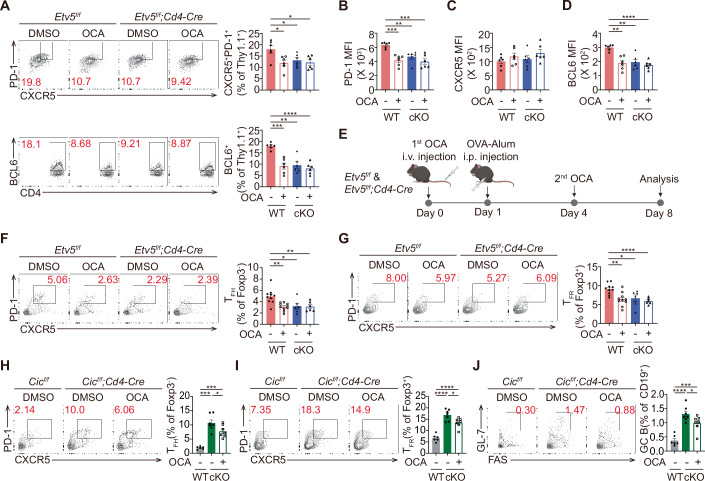
Obeticholic acid (OCA) restrains T follicular helper (T_FH_) cell differentiation via ETV5. (**A**–**D**) Flow cytometric analysis of CXCR5^+^PD-1^+^ cells and BCL6^+^ cells (**A**), PD-1 mean fluorescence intensity (MFI; **B**), CXCR5 MFI (**C**), and BCL6 MFI (**D**) generated from *Etv5*^*f/f*^ (wild-type; WT) or *Etv5*^*f/f*^*;Cd4-Cre* (cKO) OT-II cells cultured under T_FH_-polarizing conditions in the presence of dimethyl sulfoxide (DMSO; vehicle) or OCA (50 μM) for 3 days (*n* = 6 per group). Data are from a single experiment. (**E**–**G**) *Etv5*^*f/f*^ (WT) and *Etv5*^*f/f*^*;Cd4-Cre* (cKO) mice were immunized with ovalbumin (OVA) in alum and intravenously administered DMSO (vehicle) or OCA twice, over 8 days. (**E**) Schematic of the experimental design. Flow cytometric analysis of splenic T_FH_ (**F**) and follicular regulatory T (T_FR_) (**G**) cells from WT (*n* = 10) and cKO (*n* = 6) mice. Data are representative of three independent experiments. (**H**–**J**) Twenty-four-week-old *Cic*^*f/f*^ (WT) and *Cic*^*f/f*^*;Cd4-Cre* (cKO) mice were intravenously administered DMSO (vehicle) or OCA every 3 days for 27 days. Splenic T_FH_ cells (**H**), T_FR_ cells (**I**), and germinal center (GC) B cells (**J**) were analyzed via flow cytometry (WT, *n* = 6; cKO, *n* = 7). Data are representative of three independent experiments. Bar graphs present the data as mean ± SEM values. Statistical significance was assessed using two-tailed unpaired Student’s *t* tests for the indicated pairwise comparisons. Each dot represents an individual mouse. *P < 0.05, **P < 0.01, ***P < 0.001, and ****P < 0.0001. Exact *P* values are provided in Appendix Table S1. Source data are available online for this figure.

**Figure EV1:**
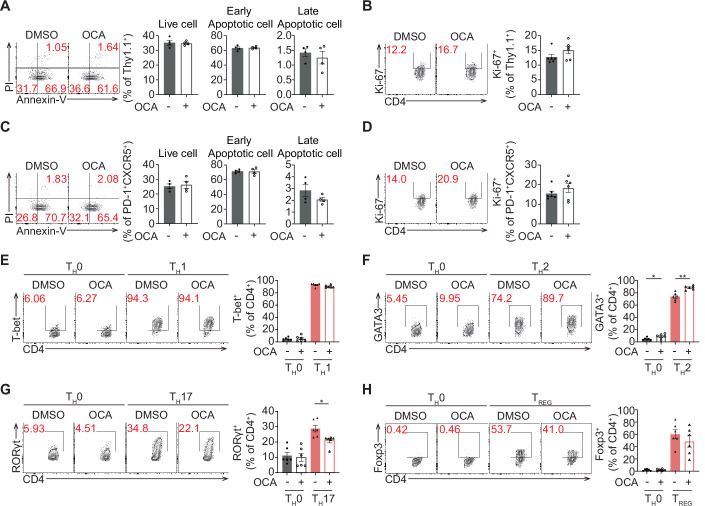
Effect of obeticholic acid (OCA) treatment on survival, proliferation, and differentiation of CD4^+^ T cells in vitro. (**A**) Flow cytometric analysis of apoptosis in Thy1.1^+^ WT OT-II cells cultured under T follicular helper (T_FH_)-polarizing conditions in the presence of dimethyl sulfoxide (DMSO) or OCA. Frequencies of live (PI^−^Annexin V^−^), early apoptotic (PI^−^Annexin V^+^), and late apoptotic (PI^+^Annexin V^+^) cells were quantified (*n* = 4 per group). Data are representative of two independent experiments. (**B**) Flow cytometric analysis of Ki-67^+^ Thy1.1^+^ WT OT-II cells after 3 days of T_FH_-polarizing culture in the presence of DMSO or OCA (*n* = 6 per group). Data are representative of three independent experiments. (**C**) Flow cytometric analysis of apoptosis in CXCR5^+^PD-1^+^ WT OT-II cells cultured under T_FH_-polarizing conditions in the presence of DMSO or OCA. Frequencies of live, early apoptotic, and late apoptotic cells were quantified (*n* = 4 per group). Data are representative of two independent experiments. (**D**) Flow cytometric analysis of Ki-67^+^ CXCR5^+^PD-1^+^ WT OT-II cells after 3 days of T_FH_-polarizing culture in the presence of DMSO or OCA (*n* = 6 per group). Data are representative of three independent experiments. (**E**–**H**) In vitro T helper-cell differentiation assays. Naive CD4^+^ T cells were cultured under T_H_1- (**E**), T_H_2- (**F**), T_H_17- (**G**), or T_REG_ (**H**)-polarizing conditions, in the presence of DMSO or OCA. Data are representative of two independent experiments. Bar graphs present the data as mean ± SEM values. Statistical significance was determined using two-tailed unpaired Student’s *t* tests for the indicated pairwise comparisons. Each dot represents a biological replicate. *P < 0.05 and **P < 0.01. Exact *P* values are in Appendix Table S1. Source data are available online for this figure.

To determine the effect of OCA on T_FH_ cell differentiation, we performed an in vitro murine T_FH_ differentiation assay using wild-type (WT) and *Etv5*-deficient Thy1.1^+^ OT-II cells (Gao et al, 2020; Park et al, 2024). As expected, *Etv5*-null OT-II cells exhibited markedly reduced T_FH_ cell differentiation compared with WT cells (Fig. 1A) (Park et al, 2024). Notably, OCA treatment suppressed T_FH_ cell differentiation in WT OT-II cells to levels comparable to those in *Etv5*-deficient cells, primarily through downregulation of PD-1 and BCL6, whereas it had no such effect in *Etv5*-deficient OT-II cells (Fig. 1A–D). The frequencies of apoptotic and proliferating (Ki-67^+^) cells within the total OT-II or PD-1^+^CXCR5^+^ population were unaffected by OCA treatment (Fig. EV1A–D), demonstrating that OCA does not impair cell viability or proliferation. To assess whether OCA affects other CD4^+^ T-cell subsets, we conducted in vitro differentiation assays for T_H_1, T_H_2, T_H_17, and regulatory T (T_REG_) cells. OCA did not significantly alter T_H_1 or T_REG_ cell differentiation (Fig. EV1E,H). T_H_2 differentiation was modestly enhanced upon OCA treatment, whereas T_H_17 differentiation was reduced (Fig. EV1F,G).

**Figure EV2:**
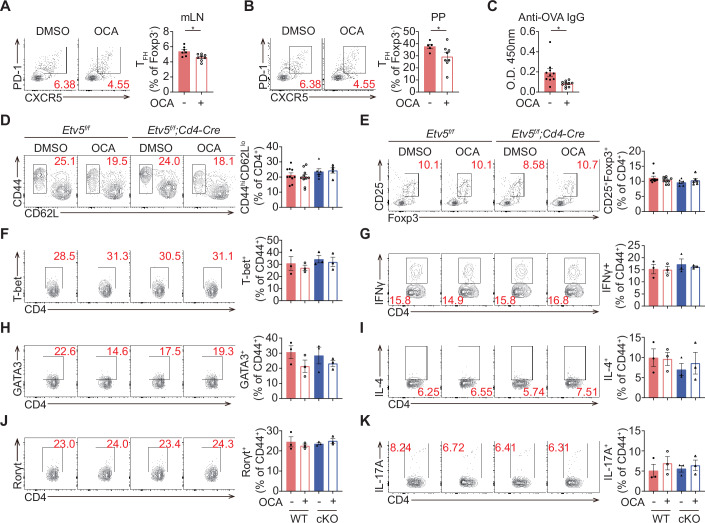
Effect of obeticholic acid (OCA) treatment on T_FH_ and CD4^+^ T cell subsets after ovalbumin (OVA) immunization. (**A**) Flow cytometric analysis of CXCR5^+^PD-1^+^ T_FH_ cells in the mesenteric lymph nodes (mLN) from mice immunized with OVA in alum and treated with dimethyl sulfoxide (DMSO; *n* = 7) or OCA (*n* = 8). Data are representative of two independent experiments. (**B**) Flow cytometric analysis of CXCR5^+^PD-1^+^ T_FH_ cells in the Peyer’s patches (PP) from mice immunized with OVA in alum and treated with DMSO (*n* = 5) or OCA (*n* = 8). Data are representative of two independent experiments. (**C**) ELISA of serum anti-OVA IgG levels in mice immunized with OVA in alum and treated with DMSO or OCA (*n* = 10 per group). Data are representative of two independent experiments. (**D**, **E**) Flow cytometric analysis of CD4^+^ T cell subsets in OVA-immunized *Etv5*^*f/f*^ (WT) and *Etv5*^*f/f*^*;Cd4-Cre* (cKO) mice treated with DMSO or OCA. Shown are the frequencies of effector CD4^+^ T cells (CD44^hi^CD62L^lo^) (**D**), T_REG_ cells (CD25^+^Foxp3^+^) (**E**) from WT (*n* = 10) and cKO (*n* = 6) mice. Data are representative of three independent experiments. (**F**–**K**) Flow cytometric analysis of CD4^+^ T cell subsets in OVA-immunized *Etv5*^*f/f*^ (WT) and *Etv5*^*f/f*^*;Cd4-Cre* (cKO) mice treated with DMSO or OCA. Shown are the frequencies of T_H_1 cells (T-bet^+^ or IFN-γ^+^) (**F**, **G**), T_H_2 cells (GATA3^+^ or IL-4^+^) (**H**, **I**), and T_H_17 cells (Rorγt^+^ or IL-17a^+^) (**J**, **K**) (*n* = 3 per group). Data are from a single experiment. Bar graphs present the data as mean ± SEM values. Statistical significance was determined using two-tailed unpaired Student’s *t* tests for the indicated pairwise comparisons. Each dot represents an individual mouse. *P < 0.05. Exact *P* values are provided in Appendix Table S1. Source data are available online for this figure.

We next examined the effect of OCA in vivo. *Etv5*^*f/f*^ (WT) and T cell-specific *Etv5*-null (*Etv5*^*f/f*^*;Cd4-Cre*) mice were administered OCA intravenously, one day before immunization with ovalbumin (OVA) in alum, followed by a second OCA injection 3 days later (Fig. 1E). Mice were analyzed on day 7 post-immunization (Fig. 1E). In WT mice, OCA treatment significantly reduced the frequencies of splenic T_FH_ and follicular regulatory T (T_FR_) cells (Fig. 1F,G). Besides the spleen, OCA also decreased T_FH_ cell frequencies in the mesenteric lymph nodes and Peyer’s patches (Fig. EV2A,B). Consistent with the reduction in the T_FH_ cell frequency, OCA-treated mice exhibited significantly reduced OVA-specific IgG levels (Fig. EV2C). As previously reported (Park et al, 2024), *Etv5*^*f/f*^*;Cd4-Cre* mice displayed reduced frequencies of T_FH_ and T_FR_ cells compared with *Etv5*^*f/f*^ mice (Fig. 1F,G). Importantly, unlike in *Etv5*^*f/f*^ mice, OCA did not reduce the T_FH_ and T_FR_ cell frequencies in *Etv5*^*f/f*^*;Cd4-Cre* mice (Fig. 1F,G), which indicated that OCA inhibits T_FH_ cell differentiation in an ETV5-dependent manner. Other CD4^+^ T-cell subsets, including effector CD4^+^ T cells, T_REG_, T_H_1, T_H_2, and T_H_17 cells, were unaffected by OCA treatment, which was suggestive of a selective effect of OCA on T_FH_ cell differentiation in vivo (Fig. EV2D–K).

**Figure EV3:**
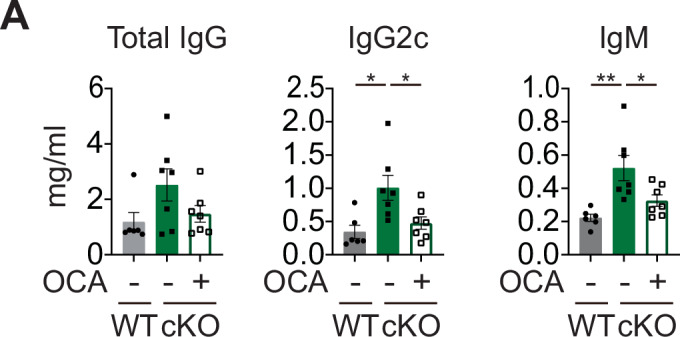
Effect of obeticholic acid (OCA) treatment on serum immunoglobulin levels in T-cell-specific CIC-deficient mice. (**A**) ELISA of serum levels of total IgG, IgG2c, and IgM in *Cic*^*f/f*^ (wild-type; WT) and *Cic*^*f/f*^*;Cd4-Cre* (cKO) mice treated with dimethyl sulfoxide (DMSO) or OCA (WT, *n* = 6 and cKO, *n* = 7). Data are representative of three independent experiments. Bar graphs present the data as mean ± SEM values. Statistical significance was determined using two-tailed unpaired Student’s *t* tests for the indicated pairwise comparisons. Each dot represents an individual mouse. *P < 0.05 and **P < 0.01. Exact *P* values are provided in Appendix Table S1. Source data are available online for this figure.

To further validate the modulation of T_FH_ cell differentiation by OCA, we examined its effect in T cell-specific capicua (CIC) knockout (*Cic*^*f/f*^*;Cd4-Cr*e) mice, which exhibit ETV5 derepression, enhanced T_FH_ cell differentiation, and lupus-like features (Park et al, 2017). Six-month-old *Cic*^*f/f*^*;Cd4-Cr*e mice received OCA intravenously every 3 days for a total of 10 doses. Consistent with previous findings (Park et al, 2017), frequencies of T_FH_, T_FR_, and GC B cells were increased in *Cic*^*f/f*^*;Cd4-Cr*e mice compared with those in control *Cic*^*f/f*^ mice (Fig. 1H–J). OCA treatment partially, but significantly, reduced both T_FH_ and T_FR_ cell frequencies and concomitantly decreased GC B cell frequency in *Cic*^*f/f*^*;Cd4-Cr*e mice (Fig. 1H–J), which was indicative of the attenuation of GC responses by OCA-mediated suppression of T_FH_ cell differentiation. Moreover, OCA treatment significantly reduced serum IgG2c and IgM levels, whereas total IgG showed a decreasing trend that did not reach statistical significance; notably, IgG2c levels decreased to a range comparable to that in WT mice (Fig. EV3A). Together, these data suggest that OCA partially suppresses T_FH_-associated responses in ETV5 derepression settings.

### OCA suppresses T_FH_ cell differentiation by disrupting the ETV5–SPP1 axis in mice and humans

**Figure 2:**
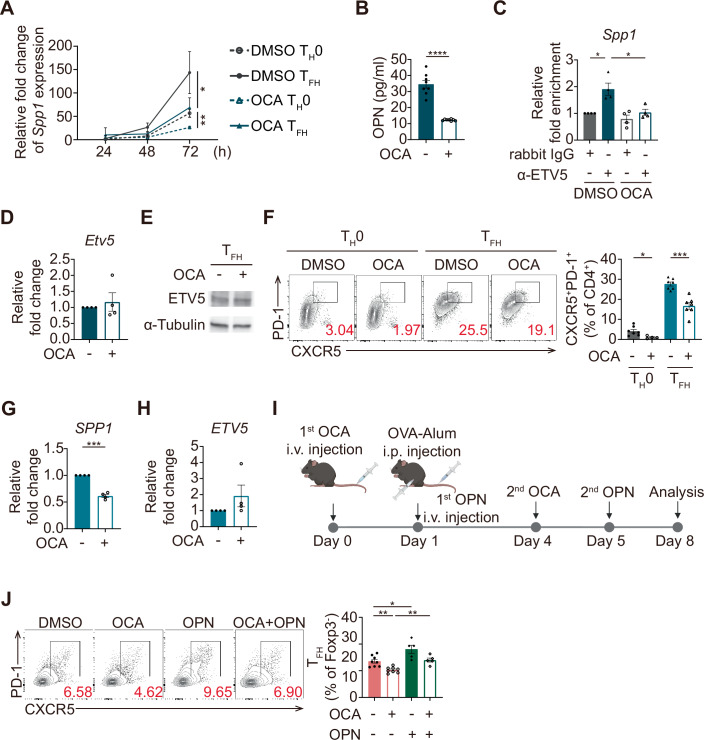
Obeticholic acid (OCA) suppresses T follicular helper (T_FH_) cell differentiation by inhibiting the ETV5–SPP1 axis. (**A**) Real-time quantitative polymerase chain reaction (RT-qPCR) analysis of *Spp1* expression in mouse naive CD4^+^ T cells cultured under T_H_0- or T_FH_-polarizing conditions in the presence of dimethyl sulfoxide (DMSO) or OCA (50 μM), for 24, 48, or 72 h (*n* = 4 per group). Data are representative of two independent experiments. (**B**) Enzyme-linked immunosorbent assay (ELISA) of osteopontin (OPN) levels in culture supernatants from mouse naive CD4^+^ T cells cultured under T_FH_-polarizing conditions in the presence of DMSO or OCA, for 72 h (*n* = 8 per group, biological replicates). Data are representative of two independent experiments. (**C**) Chromatin immunoprecipitation-quantitative polymerase chain reaction (ChIP–qPCR) analysis of ETV5 binding to the *Spp1* promoter in mouse naive CD4^+^ T cells cultured under T_FH_-polarizing conditions in the presence of DMSO or OCA. qPCR was performed for *Spp1* promoter regions containing the canonical ETV5 binding motif. Data are representative of four independent experiments. (**D**) RT-qPCR analysis of *Etv5* expression in mouse naive CD4^+^ T cells cultured under T_FH_-polarizing conditions in the presence of DMSO or OCA, for 72 h (*n* = 4 per group). Data are representative of two independent experiments. (**E**) Western blot analysis of ETV5 protein levels in mouse naive CD4^+^ T cells cultured under T_FH_-polarizing conditions in the presence of DMSO or OCA for 3 days. Data are representative of three independent experiments. (**F**) Flow cytometric analysis of human naive CD4^+^ T cells isolated from independent tonsil donors and cultured under T_H_0- or T_FH_-polarizing conditions in the presence of DMSO or OCA for 3 days (T_H_0 without OCA, *n* = 7; T_H_0 with OCA, *n* = 4; T_FH_ with or without OCA, *n* = 7). Data are representative of three independent experiments. (**G**, **H**) RT-qPCR analysis of *SPP1* (**G**) and *ETV5* (**H**) expression in human naive CD4^+^ T cells cultured under T_H_0- or T_FH_-polarizing conditions, in the presence of DMSO or OCA (50 μM), for 3 days (*n* = 4 per group). Data are representative of three independent experiments. (**I**, **J**) Mice were immunized with OVA in alum and intravenously treated with DMSO or OCA, together with phosphate-buffered saline (PBS) or OPN (3 μg), as indicated. (**I**) Schematic of the experimental design. (**J**) Flow cytometric analysis of splenic T_FH_ cells (DMSO, *n* = 8; OCA, *n* = 8; OPN, *n* = 5; OCA + OPN, *n* = 5). Data are representative of two independent experiments. Bar graphs present the data as mean ± SEM values. Statistical significance was determined using two-tailed unpaired Student’s *t* tests for the indicated pairwise comparisons. Each dot represents a biological replicate. *P < 0.05, **P < 0.01, ***P < 0.001, and ****P < 0.0001. Exact *P* values are provided in Appendix Table S1. Source data are available online for this figure.

Because ETV5 promotes T_FH_ cell differentiation by inducing the expression of *SPP1*, which encodes OPN (Park et al, 2024), we examined whether OCA suppresses *Spp1* expression during T_FH_ differentiation. *Spp1* expression was analyzed in naive mouse CD4^+^ T cells cultured under T_FH_-polarizing conditions (IL-6, IL-21, anti-IL-4, anti-IFN-γ, and anti-TGF-β), with or without OCA, for 3 days. As previously reported (Park et al, 2024), *Spp1* expression was markedly upregulated under T_FH_-polarizing conditions compared with that under T_H_0 conditions and increased progressively (Fig. 2A). OCA treatment significantly reduced *Spp1* expression to near baseline T_H_0 levels (Fig. 2A). Consistent with this, OPN secretion was markedly diminished in OCA-treated cells cultured under T_FH_-polarizing conditions at 72 h, the time point corresponding to maximal *Spp1* expression (Fig. 2B).

Given that OCA suppresses ETV5 activity by inhibiting its DNA binding (Lee et al, 2025), we next examined whether OCA interferes with ETV5 occupancy at the *Spp1* promoter. Chromatin from cells cultured under T_FH_-polarizing conditions, with or without OCA, was subjected to chromatin immunoprecipitation (ChIP) using an anti-ETV5 antibody, followed by quantitative PCR. OCA treatment substantially reduced ETV5 binding to the *Spp1* promoter region containing the canonical ETV5 motif [5′-(C/G)(C/A)GGA(A/T)(G/C)(T/C)(G/A)-3′] (Fig. 2C). In contrast, *Etv5* mRNA and ETV5 protein levels remained unchanged upon OCA treatment (Fig. 2D,E), consistent with previous observations (Lee et al, 2025). These data indicate that OCA suppresses T_FH_ cell differentiation by blocking ETV5 binding to the *Spp1* promoter, thereby repressing *Spp1* expression.

We next investigated whether OCA exerts a similar inhibitory effect on T_FH_ differentiation in humans. Naive human CD4^+^ T cells were cultured for 3 days under T_FH_-polarizing (anti-CD3/CD28 plus IL-12 and TGF-β) or T_H_0 conditions in the presence or absence of OCA. OCA treatment significantly inhibited T_FH_ differentiation under both T_H_0 and T_FH_ conditions (Fig. 2F). Consistent with our murine data, OCA also significantly downregulated *SPP1* expression in human CD4^+^ T cells cultured under T_FH_-polarizing conditions (Fig. 2G), whereas *ETV5* expression remained unaffected (Fig. 2H). These findings suggest that OCA suppresses T_FH_ cell differentiation in both mouse and human CD4^+^ T cells by inhibiting the conserved ETV5–SPP1 axis.

To further examine whether the effect of OCA is mediated via suppression of the ETV5–SPP1 axis in vivo, we performed an OPN rescue experiment. Mice were administered OCA intravenously 1 day before immunization with OVA in alum. The next day, recombinant OPN was injected intravenously, followed 2 h later by intraperitoneal immunization with OVA in alum. A second dose of OCA and OPN was administered 3 and 4 days after the initial treatment, respectively, and mice were analyzed on day 7 after immunization (Fig. 2I). As previously reported (Park et al, 2024), OPN treatment markedly increased T_FH_ cell frequency compared with that in the control group (Fig. 2J). Notably, mice treated with both OCA and OPN exhibited higher T_FH_ cell frequencies than those treated with OCA alone, with the levels reaching those in control mice (Fig. 2J). These results indicate that recombinant OPN restores T_FH_ cell differentiation in OCA-treated mice, further supporting the notion that the inhibitory effect of OCA on T_FH_ differentiation is mediated through suppression of the ETV5–SPP1 axis.

### OCA alleviates disease symptoms in pristane-induced lupus mouse models in an ETV5-dependent manner

**Figure 3:**
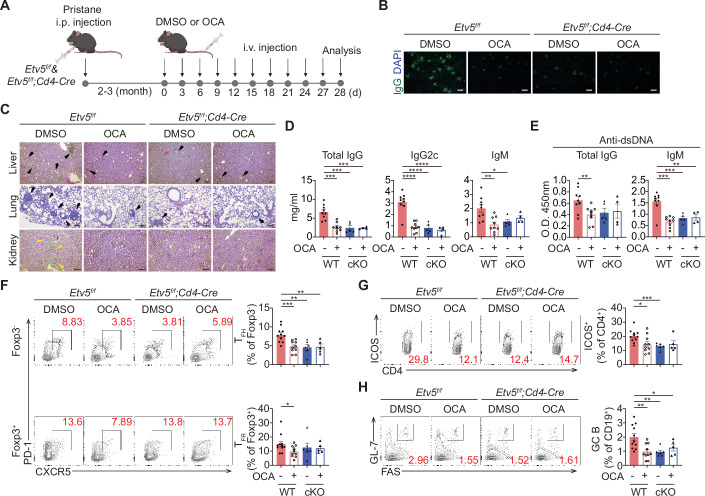
Therapeutic effect of obeticholic acid (OCA) in pristane-induced lupus mouse models. (**A**) Schematic illustration of the experimental design. Two to three months after pristane injection, *Etv5*^*f/f*^ (WT) and *Etv5*^*f/f*^*;Cd4*-*Cre* (cKO) mice were intravenously administered dimethyl sulfoxide (DMSO; vehicle), or OCA, followed by dosing every 3 days for 27 days. (**B**) Immunofluorescence images showing IgG deposition in the kidney glomeruli of wild-type (WT) and cKO mice treated with pristane for 2–3 months, followed by treatment with DMSO or OCA. The kidney sections were stained with anti-IgG (green) and DAPI (blue). Data are representative of four independent experiments. Scale bar, 100 μm. (**C**) Hematoxylin and eosin staining of the liver, lung, and kidney sections from WT and cKO mice treated with pristane for 2–3 months, followed by treatment with DMSO or OCA. Black arrows indicate immune cell infiltration; yellow arrows indicate glomerular abnormalities. Data are representative of four independent experiments. Scale bar, 100 μm. (**D**, **E**) Enzyme-linked immunosorbent assay (ELISA) for serum levels of total IgG, IgG2c, and IgM (**D**), and of total IgG and IgM anti-dsDNA antibodies (**E**) in WT and cKO mice treated with DMSO or OCA (WT DMSO, *n* = 9; WT OCA, *n* = 9; cKO DMSO, *n* = 5; cKO OCA, *n* = 4). Data are representative of five independent experiments. (**F**–**H**) Flow cytometric analysis of splenic T follicular helper (T_FH_) and follicular regulatory T (T_FR_) cells (**F**), ICOS^+^ T cells (**G**), and germinal center (GC) B cells (**H**) in WT and cKO mice treated with DMSO or OCA (WT DMSO, *n* = 11; WT OCA, *n* = 10; cKO DMSO, *n* = 6; cKO OCA, *n* = 5). Data are representative of five independent experiments. Bar graphs present the data as mean ± SEM values. Statistical significance was determined using two-tailed unpaired Student’s *t* tests for the indicated pairwise comparisons. Each dot represents an individual mouse. *P < 0.05, **P < 0.01, ***P < 0.001, and ****P < 0.0001. Exact *P* values are provided in Appendix Table S1. Source data are available online for this figure.

Given the ability of OCA to suppress T_FH_ cell differentiation, we next evaluated its therapeutic potential in mouse models of lupus. WT and *Etv5*^*f/f*^*;Cd4-Cr*e mice were intraperitoneally injected with tetramethylpentadecane (pristane) (Park et al, 2024; Reeves et al, 2009) and, after 2–3 months, intravenously administered dimethyl sulfoxide (DMSO) or OCA every 3 days for a total of 27 days (Fig. 3A). As reported previously (Park et al, 2024), DMSO-treated *Etv5*^*f/f*^*;Cd4-Cr*e mice exhibited attenuated pristane-induced lupus phenotypes compared with WT controls, characterized by reduced IgG deposition in the kidney glomeruli, amelioration of histopathological abnormalities, and lower serum immunoglobulin and anti-dsDNA autoantibody levels (Fig. 3B–E; Appendix Fig. S1A–C). Notably, OCA treatment markedly alleviated pristane-induced lupus phenotypes in WT mice, whereas it did not confer additional therapeutic benefits in *Etv5*^*f/f*^*;Cd4-Cr*e mice (Fig. 3B–E; Appendix Fig. S1A–C), indicating that the therapeutic effect of OCA is predominantly ETV5-dependent.

**Figure EV4:**
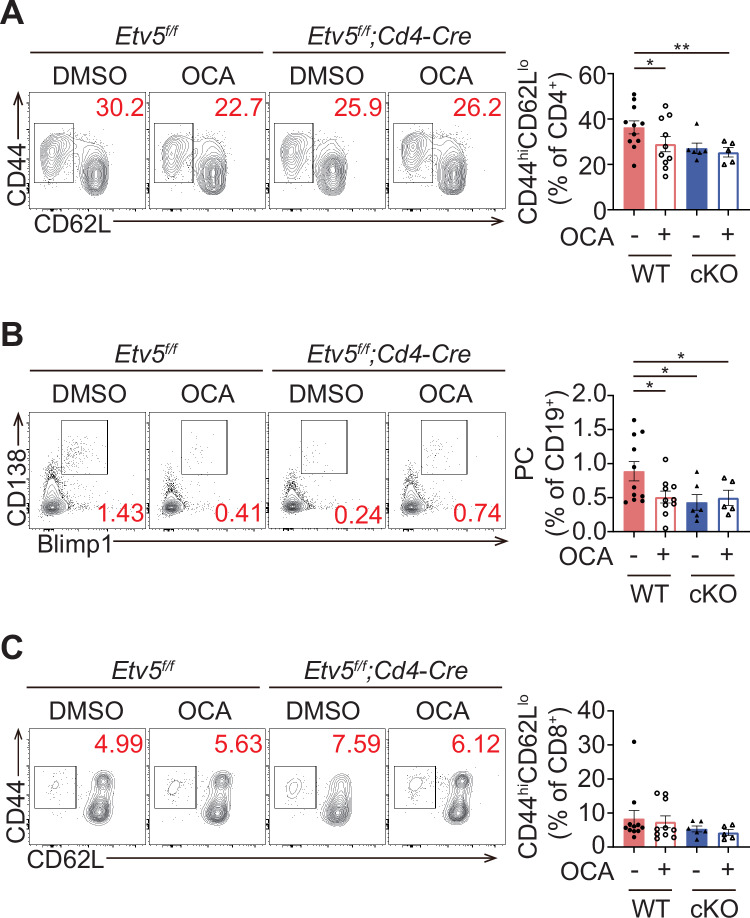
Effects of obeticholic acid (OCA) on effector T-cell and plasma-cell differentiation in pristane-induced lupus mouse model. (**A**–**C**) Flow cytometric analysis of T cells and plasma cells in *Etv5*^*f/f*^ (wild-type; WT) and *Etv5*^*f/f*^*;Cd4-Cre* (cKO) mice treated with pristane and subsequently administered dimethyl sulfoxide (DMSO) or OCA. Shown are the frequencies of CD4^+^ effector T cells (CD44^hi^CD62L^lo^) (**A**), CD138^+^Blimp1^+^ plasma cells (**B**), and CD8^+^ effector T cells (CD44^hi^CD62L^lo^) (**C**) (WT DMSO, *n* = 11; WT OCA, *n* = 10; cKO DMSO, *n* = 6; cKO OCA, *n* = 5). Data are representative of five independent experiments. Bar graphs present the data as mean ± SEM values. Statistical significance was determined using two-tailed unpaired Student’s *t* tests for the indicated pairwise comparisons. Each dot represents an individual mouse. *P < 0.05 and **P < 0.01. Exact *P* values are provided in Appendix Table S1. Source data are available online for this figure.

Flow cytometric analysis of WT mice showed that OCA treatment significantly reduced the frequencies of T_FH_, GC B, effector CD4^+^ T, and plasma cells, but had no effect on effector CD8^+^ T cells (Figs. 3F–H and EV4A–C). Consistent with previous findings (Fig. 1F, G), OCA treatment did not further suppress these populations in *Etv5*^*f/f*^*;Cd4-Cr*e mice (Figs. 3F–H and EV4A,B). Taken together, these results indicate that the therapeutic effect of OCA in pristane-induced lupus is largely ETV5-dependent and is associated with suppression of T_FH_ cell-driven humoral immune responses.

### OCA ameliorates lupus pathogenesis by targeting the ETV5–SPP1 axis in T_FH_ cells

**Figure 4:**
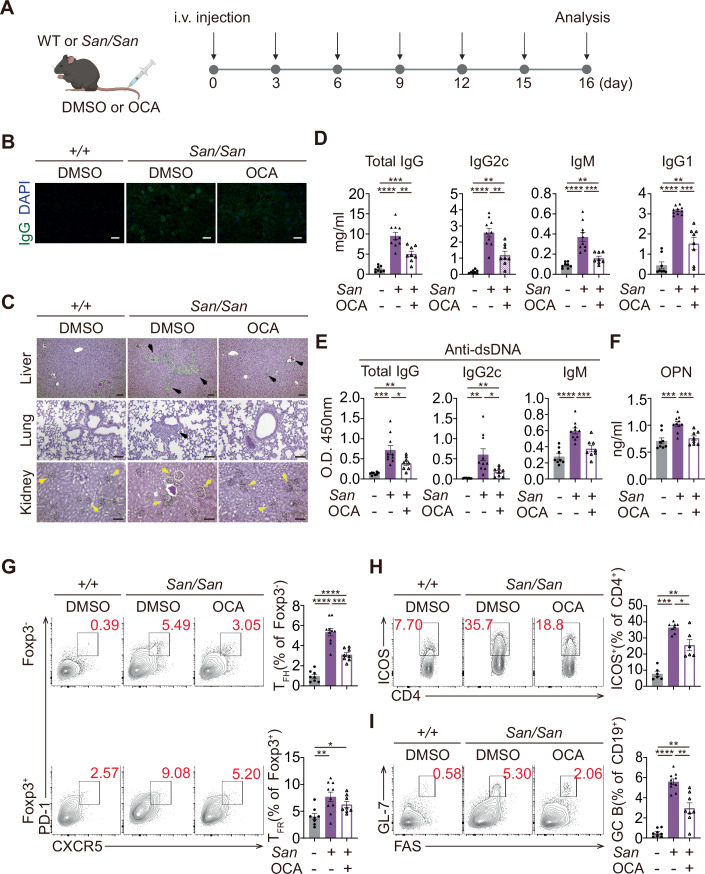
Therapeutic efficacy of obeticholic acid (OCA) in *Sanroque* mice. (**A**) Schematic illustration of the experimental design. Seven-week-old wild-type (WT) and *Sanroque* mice were intravenously administered dimethyl sulfoxide (DMSO, vehicle) or OCA every 3 days for 16 days. (**B**) Immunofluorescence images of IgG deposition in the kidney glomeruli from WT and *Sanroque* mice treated with DMSO or OCA. The kidney sections were stained with anti-IgG (green) and DAPI (blue). Data are representative of four independent experiments. Scale bar, 100 μm. (**C**) Hematoxylin and eosin staining of the liver, lung, and kidney sections from WT and *Sanroque* mice treated with DMSO or OCA. Black arrows indicate immune cell infiltration; yellow arrows indicate glomerular abnormalities. Data are representative of two independent experiments. Scale bar, 100 μm. (**D**, **E**) ELISA for serum levels of total IgG, IgG2c, IgM, and IgG1 (**D**), and of anti-dsDNA antibodies (**E**) in WT mice treated with DMSO (*n* = 8) and *Sanroque* mice treated with DMSO (*n* = 10) or OCA (*n* = 8). Data are representative of four independent experiments. (**F**) ELISA of serum osteopontin (OPN) levels in WT mice treated with DMSO (*n* = 8) and *Sanroque* mice treated with DMSO (*n* = 10) or OCA (*n* = 8). Data are representative of four independent experiments. (**G**–**I**) Flow cytometric analysis of splenic T follicular helper (T_FH_) and follicular regulatory T (T_FR_) cells (**G**), ICOS^+^ T cells (**H**), and germinal center (GC) B cells (**I**) in WT mice treated with DMSO (*n* = 8) and *Sanroque* mice treated with DMSO (*n* = 10) or OCA (*n* = 8). Data are representative of five independent experiments. Bar graphs present the data as mean ± SEM values. Statistical significance was determined using two-tailed unpaired Student’s *t* tests for the indicated pairwise comparisons. Each dot represents an individual mouse. *P < 0.05, **P < 0.01, ***P < 0.001, and ****P < 0.0001. Exact *P* values are provided in Appendix Table S1. Source data are available online for this figure.

**Figure EV5:**
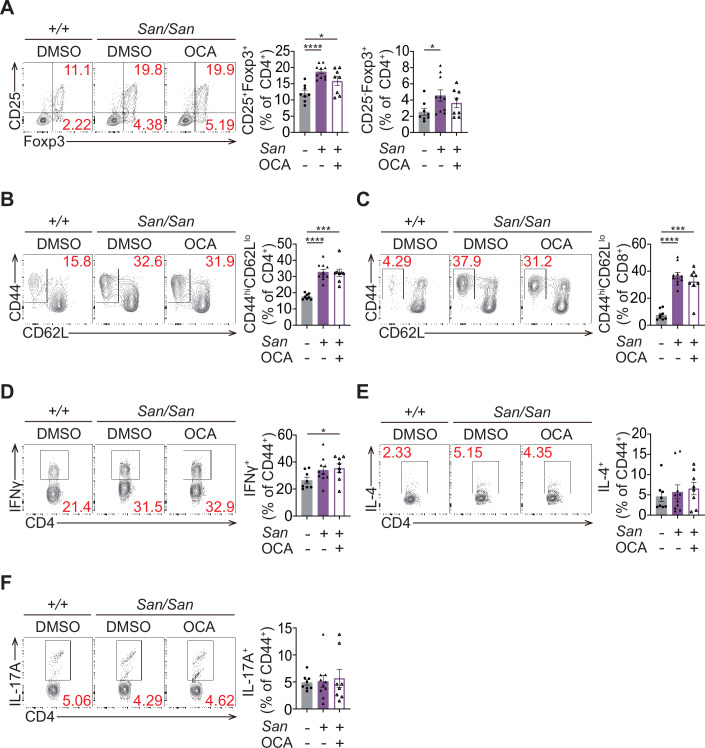
Effects of obeticholic acid (OCA) on T-cell activation and differentiation in *Sanroque* mice. (**A**–**F**) Flow cytometric analysis of T-cell subsets in wild-type (WT) and *Sanroque* mice treated with dimethyl sulfoxide (DMSO) or OCA. Shown are the frequencies of T_REG_ cells (CD25^+^Foxp3^+^ and CD25^−^Foxp3^+^) (**A**), CD4^+^ effector T cells (CD44^hi^CD62L^lo^) (**B**), CD8^+^ effector T cells (CD44^hi^CD62L^lo^) (**C**), T_H_1 cells (IFN-γ^+^) (**D**), T_H_2 cells (IL-4^+^) (**E**), and T_H_17 cells (IL-17a^+^) (**F**). WT mice were treated with DMSO (*n* = 8) and Sanroque mice were treated with either DMSO (*n* = 10) or OCA (*n* = 8). Data are representative of five independent experiments. Bar graphs present the data as mean ± SEM values. Statistical significance was determined using two-tailed unpaired Student’s *t* tests for the indicated pairwise comparisons. Each dot represents an individual mouse. *P < 0.05, ***P < 0.001, and ****P < 0.0001. Exact *P* values are provided in Appendix Table S1. Source data are available online for this figure.

To verify the therapeutic efficacy of OCA in T_FH_ cell-driven pathogenesis of lupus, we evaluated its effect in the *Sanroque* spontaneous lupus model, which is characterized by excessive T_FH_ cell formation due to the *roquin-1* R199M mutation (Park et al, 2024; Vinuesa et al, 2005). Seven-week-old WT and *Sanroque* mice were treated with OCA every 3 days for a total of 16 days (Fig. 4A). OCA treatment substantially ameliorated the lupus-like phenotype of *Sanroque* mice, including reduced IgG deposition and attenuated histopathological abnormalities, approaching WT levels (Fig. 4B,C; Appendix Fig. S2A–C). Serum immunoglobulin and anti-dsDNA autoantibody levels were also significantly decreased, but they were not fully restored to WT levels (Fig. 4D, E). Given the reported association of dysregulated OPN expression with autoimmune diseases, including rheumatoid arthritis and SLE (Rullo et al, 2013; Wong et al, 2005; Xu et al, 2022) and our finding of the ETV5–SPP1 axis contributing to OCA-mediated inhibition of T_FH_ cell differentiation, we next measured serum OPN levels in this model. Serum OPN levels were increased in *Sanroque* mice relative to those in WT mice, and OCA treatment reduced them to near-baseline WT levels (Fig. 4F). Flow cytometric analysis further showed that OCA treatment significantly decreased the frequencies of T_FH_ and GC B cells (Fig. 4G–I) but did not significantly alter the CD25^+^ T_REG_, CD25^−^ T_REG_, effector CD4^+^ T, effector CD8^+^ T, T_H_1, T_H_2, and T_H_17 cell populations (Fig. EV5A–F). Together, these results suggest that OCA alleviates lupus-like pathology by suppressing ETV5-driven OPN expression and restraining T_FH_ cell-dependent immune responses.

## Discussion

In this study, we identified the transcription factor ETV5 as a druggable checkpoint in T_FH_ cell differentiation and demonstrated that OCA suppresses the T_FH_–GC axis and ameliorates lupus pathogenesis by inhibiting the ETV5–SPP1 axis. Although aberrant T_FH_ cell expansion is the central driver of GC responses and pathogenic autoantibody production in systemic autoimmune diseases such as SLE (Blanco et al, 2016; Christodoulou et al, 2025; Nakayamada and Tanaka, 2021), therapeutic approaches that directly target this pathway remain limited. Our findings that OCA reduces T_FH_-cell differentiation in both mouse and human CD4^+^ T cells support its translational potential in SLE. Notably, the selective reduction of T_FH_ and GC B cell populations by OCA, while sparing other CD4^+^ T helper subsets, underscores its cell-type specificity. This selective immunomodulatory effect of OCA contrasts with those of conventional immunosuppressive agents, which broadly dampen immune responses and often cause systemic toxicity (He and Li, 2023; Katarzyna et al, 2023; Popa et al, 2018). Thus, inhibition of ETV5 activity by OCA may represent a promising strategy to minimize treatment-associated toxicity in SLE.

The therapeutic potential of OCA was further supported across two complementary lupus models. In both the pristane-induced and spontaneous *Sanroque* models, OCA treatment consistently reduced autoantibody titers, immune complex deposition, and lupus-associated tissue pathology. These findings suggest that modulation of the ETV5–SPP1 axis can mitigate autoimmune pathology across distinct disease contexts. Given that OCA is an FDA-approved drug for PBC, with an established clinical safety profile (Ali and Lindor, 2016), our results provide a strong rationale for repurposing OCA in T_FH_ cell-driven systemic autoimmunity.

The receptor specificity underlying the immunomodulatory effect of OCA in T cells is an important unresolved issue. OCA is a well-established agonist of FXR (Ali and Lindor, 2016); recent studies have suggested that FXR signaling promotes pathogenic T_FH_ cell responses in lupus (Wang et al, 2025). This raises an important question as to how OCA suppresses T_FH_ cell differentiation. Our data show that OCA suppresses T_FH_ cell differentiation by inhibiting ETV5 binding to the *Spp1* promoter without altering ETV5 expression levels, supporting a mechanism centered on disruption of ETV5 transcriptional activity. Moreover, the absence of an additional inhibitory effect under *Etv5*-deficient conditions further supports ETV5 as a key mediator of OCA-dependent suppression of T_FH_ cell differentiation. Nonetheless, the possibility that FXR-dependent signaling also contributes to this process cannot be excluded. Dissecting FXR-dependent versus ETV5-specific pathways will therefore be important to fully resolve the mechanism of action of OCA in T cells.

Although our ChIP–qPCR data demonstrate reduced ETV5 binding at the *Spp1* promoter (Fig. 2C), whether OCA broadly remodels ETV5 chromatin occupancy or selectively disrupts binding at specific target loci remains to be determined. The OPN rescue experiment strongly supports the functional importance of the ETV5–SPP1 axis (Fig. 2J), but it also leaves open the possibility that additional ETV5-regulated genes contribute to the observed suppression of T_FH_ cell differentiation. Future genome-wide analysis of ETV5 chromatin occupancy will therefore be necessary to define the extent of this regulatory mechanism.

Our findings also highlight broader implications for targeting transcription factors as a therapeutic strategy. Although transcription factors have historically been considered difficult to target pharmacologically, recent advances have shown that their activity can be modulated through interference with DNA binding or cofactor interactions (Henley and Koehler, 2021). OCA provides a compelling example of this emerging paradigm by selectively disrupting the ETV5–SPP1 transcriptional axis, thereby reprogramming T_FH_ cell fate without broadly impairing immune function. This conceptual framework may be extended to other autoimmune diseases in which dysregulated transcriptional programs drive pathogenic immune responses.

In summary, our study establishes ETV5 as a tractable therapeutic node within the T_FH_–GC axis and identifies OCA as a clinically viable agent capable of restraining autoantibody-driven pathology. By demonstrating the feasibility of using a transcription factor as a selective and druggable checkpoint in T_FH_ cell differentiation, our work supports the broader concept that transcription factor-directed therapies may represent a new paradigm for precision immunomodulation in systemic autoimmunity.

## Methods

**Table:** Reagents and tools table

Reagent/resource	Reference or source	Identifier or catalog number
**Experimental models**		
Mouse: *Cic*^*fl/fl*^	Lu et al (2017)	N/A
Mouse: *Etv5*^*fl/fl*^	Zhang et al (2009)	N/A
Mouse: *Cd4-Cre*	Lee et al (2001)	N/A
Mouse: OT-II	Barnden et al (1998)	N/A
Mouse: *Sanroque*	Vinuesa et al (2005)	N/A
Human: Tonsil obtained from 3–12-year-old children	This paper	N/A
**Antibodies**		
PerCP-Cyanine5.5 CD90.1 (Thy-1.1) monoclonal antibody	eBioscience	45-0900-82; RRID: AB_2573662
PE anti-mouse CD4 antibody	BioLegend	100511; RRID: AB_312714
PerCP-Cyanine5.5 anti-mouse CD4	Tonbo Biosciences	65-0042; RRID: AB_2621876
FITC CD279 (PD-1) monoclonal antibody	eBioscience	11-9981-81; RRID: AB_465472
PE anti-mouse CD8a	eBioscience	50-0081; RRID: AB_2621741
FITC rat anti-mouse CD44	BD Biosciences	561859; RRID: AB_10894581
PE-Cy7 rat anti-mouse CD62L	BD Biosciences	560516; RRID: AB_1645257
PE anti-mouse CD25 antibody	BioLegend	102007; RRID: AB_312856
APC anti-mouse CD278 (ICOS) antibody	BioLegend	117419; RRID: AB_2832417
BV421 rat anti-mouse CD19	BD Biosciences	562701; RRID: AB_2737731
APC anti-mouse CD19 antibody	BioLegend	152409; RRID: AB_2629838
Alexa Fluor® 488 anti-mouse/human GL7 Antigen (T and B cell Activation Marker) antibody	BioLegend	144611; RRID: AB_2563284
PE anti-mouse/human GL7 Antigen (T and B cell Activation Marker) antibody	BioLegend	144607; RRID: AB_2562925
Brilliant Violet 421™ anti-mouse CD138 (Syndecan-1) antibody	BioLegend	142507; RRID: AB_11204257
Ghost Dye™ Violet 510	Tonbo Biosciences	13-0870
Biotin rat anti-mouse CD185 (CXCR5)	BD Biosciences	551960; RRID: AB_394301
Biotin Hamster anti-mouse CD95	BD Biosciences	554256; RRID: AB_395328
PE/Cyanine7 streptavidin	BioLegend	405206
AlexaFluor647 Bcl6 monoclonal antibody	BD Biosciences	561525; RRID: AB_10898007
eFlour450 FOXP3 monoclonal antibody	eBioscience	48-5773-82; RRID: AB_1518812
PE T-bet monoclonal antibody	eBioscience	12-5825-82; RRID: AB_925761
PE-Cy7 Gata-3 monoclonal antibody	eBioscience	25-9966-42; RRID: AB_2573568
APC ROR gamma (t) monoclonal antibody	eBioscience	17-6981-82; RRID: AB_2573254
PE anti-mouse Blimp-1 antibody	BioLegend	150005; RRID: AB_2565991
APC Ki-67 monoclonal antibody	eBioscience	17-5698-80; RRID: AB_2688056
APC anti-mouse IFN-γ antibody	BioLegend	505809; RRID: AB_315403
PE-Cy7 rat anti-mouse IL-4	BD Biosciences	560699; RRID: AB_1727548
PE rat anti-mouse IL-17A	BD Biosciences	559502; RRID: AB_397256
FITC anti-human CD4 antibody	BioLegend	317407; RRID: AB_571950
BV421 rat anti-human CXCR5 (CD185)	BD Biosciences	562747; RRID: AB_2737766
PE/Cyanine7 anti-human CD279 (PD-1) antibody	BioLegend	329917; RRID: AB_2159325
PerCP/Cyanine5.5 anti-human CD45RA antibody	BioLegend	304121; RRID: AB_893358
Biotin anti-mouse CD19 antibody	BioLegend	115504; RRID: AB_313639
Biotin anti-mouse/human CD45R/B220 antibody	BioLegend	103204; RRID: AB_312989
Biotin anti-mouse CD8a antibody	BioLegend	100704; RRID: AB_312743
Biotin anti-mouse/human CD11b antibody	BioLegend	101204; RRID: AB_312787
Biotin anti-mouse CD11c antibody	BioLegend	117304; RRID: AB_313773
Biotin anti-mouse CD49b (pan-NK cells) antibody	BioLegend	108904; RRID: AB_313411
Biotin anti-mouse Ly-6G/Ly-6C (Gr-1) antibody	BioLegend	108404; RRID: AB_313369
Biotin anti-mouse TER-119/erythroid cells antibody	BioLegend	116204; RRID: AB_313705
Biotin TCR gamma/delta monoclonal antibody	eBioscience	13-5811-82; RRID: AB_466684
Biotin anti-mouse/human CD44 antibody	BioLegend	103004; RRID: AB_312955
Purified NA/LE Hamster anti-mouse CD3e	BD Pharmingen	553057; RRID: AB_394590
Purified NA/LE Hamster anti-mouse CD28	BD Pharmingen	553294; RRID: AB_394763
Purified rat anti-mouse CD16/CD32 (Mouse BD Fc Block™)	BD Pharmingen	553141; RRID: AB_394656
Anti-mouse IgG (whole molecule)–FITC antibody produced in goat	Sigma-Aldrich	F0257; RRID: AB_259378
Goat anti-mouse Ig, Human ads-UNLB	Southern Biotechnology	1010-01; RRID: AB_2794121
Goat anti-mouse IgG, Human ads-HRP	Southern Biotechnology	1030-05; RRID: AB_2619742
Goat anti-mouse IgG1, Human ads-HRP	Southern Biotechnology	1070-05; RRID: AB_ 2650509
Goat anti-mouse IgG2c, Human ads-HRP	Southern Biotechnology	1079-05; RRID: AB_ 2794466
Goat anti-mouse IgM, Human ads-HRP	Southern Biotechnology	1020-05; RRID: AB_2794201
ETV5 polyclonal antibody	Proteintech	13011-1-AP; RRID: AB_2278092
*InVivo*MAb anti-mouse IL-4	BioXcell	BE0045; RRID: AB_1107707
InVivoMAb anti-mouse IFNγ	BioXcell	BE0055; RRID: AB_1107694
InVivoMAb anti-mouse/human/rat/monkey/hamster/canine/bovine TGF-β	BioXcell	BE0057; RRID: AB_1107757
Alpha tubulin antibody (A-6)	Santa Cruz Technology	sc-398103; RRID: AB_2832217
**Oligonucleotides and other sequence-based reagents**		
Mouse*_Spp1 _*Forward 5’-AGAGCGGTGAGTCTAAGGAGT-3’	Park et al (2024)	N/A
Mouse*_Spp1 _*Forward 5’-TGCCCTTTCCGTTGTTGTCC-3’	Park et al (2024)	N/A
Mouse*_Etv5*_Forward 5’-TCAGTCTGATAACTTGGTGCTTC-3′	Park et al (2024)	N/A
Mouse*_Etv5*_Reverse 5’-GGCTTCCTATCGTAGGCACAA-3'	Park et al (2024)	N/A
Mouse *Hprt* _Forward 5’-TCAGTCAACGGGGGACATAAA-3’	Park et al (2024)	N/A
Mouse *Hprt* _Reverse 5’-GGGGCTGTACTGCTTAACCAG-3’	Park et al (2024)	N/A
Human_*SPP1*_Forward 5’-CAAATACCCAGATGCTGTGGC-3’	Park et al (2024)	N/A
Human_*SPP1*_Reverse 5’-TGGTCATGGCTTTCGTTGGA-3’	Park et al (2024)	N/A
Human_*ETV5* _Forward 5’-CATCCTACATGAGAGGGGGTTA-3’	Park et al (2024)	N/A
Human_*ETV5* _Reverse 5’-AAGTATAATGGGGGATCTTTTTCA-3’	Park et al (2024)	N/A
Human_*HPRT* _Forward 5’-ACCAGTCAACAGGGGACATAA-3’	Park et al (2024)	N/A
Human_*HPRT* _Reverse 5’-CTTCGTGGGGTCCTTTTCACC-3’	Park et al (2024)	N/A
*Spp1* promoter forward 5’-AACCACAAAACCAGAGGAGG-3’	Park et al (2024)	N/A
*Spp1* promoter reverse 5’-GAGGTGGAGTGATGTGTCATG-3’	Park et al (2024)	N/A
**Chemicals, enzymes and other reagents**		
Dimethyl sulfoxide (DMSO) Cell culture grade	Applichem	A3672
Obeticholic Acid (INT-747)	Selleckchem	S7660
Recombinant Mouse Osteopontin/OPN Protein	R&D Systems	441-OP-050
Pristane	Sigma-Aldrich	P2870
EndoFit™ OVA protein	InvivoGen	vac-pova
OVA 323–339	InvivoGen	vac-isq
Recombinant Mouse IL-12 Protein	R&D Systems	419-ML-010
Mouse IL-4 Recombinant Protein	Peprotech	214-14
Mouse IL-6 Recombinant Protein	Peprotech	216-16
Mouse IL-1 beta Recombinant Protein	Peprotech	211-11B
Recombinant Human TGF-β1 (carrier-free)	Biolegend	781802
Mouse IL-2 Recombinant Protein	Peprotech	212-12
Mouse IL-21 Recombinant Protein	Peprotech	210-21
Human IL-12 p70 Recombinant Protein	Gibco	200-12H
Dynabeads™ Human T-Activator CD3/CD28 for T Cell Expansion and Activation	Gibco	11161D
Alhydrogel® adjuvant 2%	InvivoGen	vac-alu
Tissue-Tek® O.C.T. Compound	Sakura	4583
Formalin solution, neutral buffered, 10%	Sigma-Aldrich	HT501320
Glycergel Mounting Medium	Dako	C0563
Triton x-100	Sigma-Aldrich	9036-19-5
DAPI	Sigma-Aldrich	10236276001
Harris Hematoxylin Solution, Modified	Sigma-Aldrich	HHS32
Eosin Y Alcoholic	Cancer Diagnostics	EM500G
Canada balsam	Sigma-Aldrich	C1795
Mouse Reference Serum	Bethyl	RS10-101
UltraPure™ Calf Thymus DNA Solution	Invitrogen	15633019
Fetal Bovine Serum (FBS)	Welgene	S001-01
HBSS (10X), no calcium, no magnesium, no phenol red	Gibco	14185052
Lymphoprep	Stemcell Technologies	07851
RPMI 1640 Medium	Welgene	LM011-60
TMB Solution (1X)	eBioscience	00-4201-56
LPS	Sigma-Aldrich	A9543
Ionomycin from Streptomyces conglobatus	Sigma-Aldrich	I9657
PMA	Sigma-Aldrich	P1585
GolgiPlug™ Protein Transport Inhibitor	BD	555029
GolgiStop™ Protein Transport Inhibitor	BD	554724
RiboEx	GeneAll	301-002
SYBR green Real-time PCR Master Mix	TOYOBO	TOQPK-201
Protein G Agarose	Millipore	16-266
Normal Rabbit IgG	Cell Signaling Technology	2729S
Foxp3 / Transcription Factor Staining Buffer Set	eBioscience	00-5523-00
APC Annexin V Apoptosis Detection Kit with PI	BioLegend	640932
GoScript™ Reverse Transcriptase	Promega	A5004
Naive CD4 T cell isolation kit II human	Miltenyi biotec	130-094-131
EasySep™ Mouse Streptavidin RapidSpheres™ Isolation Kit	Stem Cell Technologies	19860
Mouse Osteopontin DuoSet ELISA	R&D Systems	DY441
cOmplete™ ULTRA Tablets, EDTA-free, glass vials Protease Inhibitor Cocktail	Roche	5892953001
PhosSTOP™	Roche	4906837001
Immobilon Western Chemiluminescent HRP Substrate	Merck Millipore	WBKLS0500
**Software**		
Prism version 10.5.0	GraphPad Software	https://www.graphpad.com/
FlowJo version 10.10.0	Tree Star	https://www.flowjo.com/
**Other**		
FACSymphony A5 cell analyzer	BD Biosciences	
CytoFLEX LX flow cytometer	Beckman Coulter	
ImageQuant LAS 500	GE Healthcare Life Sciences	
Olympus CKX53 Inverted Phase Contrast Microscope	Olympus	
BX43 microscope	Olympus	

### Mice

All mouse strains were maintained on a C57BL/6 background. Generation of *Cic*-floxed (Lu et al, 2017; Park et al, 2024, 2017), *Etv5*-floxed (Park et al, 2024; Zhang et al, 2009), *Cd4-Cre* (Lee et al, 2001; Park et al, 2024), OT-II (Barnden et al, 1998; Park et al, 2024), and *Sanroque* (Park et al, 2024; Vinuesa et al, 2005) mice was previously described. The analyzed mice were at the indicated stage, as mentioned in the respective figure legends. The animals were maintained in a specific pathogen-free animal facility under a standard 12-h light/12-h dark cycle. Mice were provided standard rodent chow and water ad libitum. All animal procedures were approved by the Institutional Animal Care and Use Committee of Pohang University of Science and Technology (approval no. POSTECH-2025-0083) and were performed in accordance with institutional guidelines and applicable ethical regulations.

### Pristane injection and OCA treatment

Pristane (Sigma-Aldrich) was filtered through a 0.22 μm syringe filter prior to injection. Eight-week-old *Etv5*^*f/f*^ and *Etv5*^*f/f*^*;Cd4-Cre* mice were administered a single intraperitoneal injection of pristane (0.5 mL). For OCA treatment, 2–3 months later, animals were randomly allocated to treatment groups and intravenously administered 2.5 mg/kg obeticholic acid (Selleckchem) or a vehicle solution [10% DMSO (Applichem) in phosphate-buffered saline (PBS)] every 3 days for 27 days. One day after the final injection, blood was collected from the animals prior to euthanasia. Autoimmune-like phenotypes were then assessed, and the composition of splenic immune cells was analyzed via flow cytometry.

### *Sanroque* mouse model and OCA treatment

Seven-week-old *Sanroque* and WT mice were randomly allocated to treatment groups and intravenously administered 2.5 mg/kg OCA or a vehicle solution (10% DMSO in PBS). The solutions were administered every 3 days for a total of 16 days. One day after the final injection, mice were euthanized for analysis.

### *Cic*-deficient mouse model and OCA treatment

Twenty-four-week-old *Cic*^*f/f*^ and *Cic*^*f/f*^*;Cd4-Cre* mice were randomly allocated to treatment groups and intravenously administered 2.5 mg/kg OCA or a vehicle solution (10% DMSO in PBS) every 3 days for a total of 27 days. One day after the final injection, mice were sacrificed, and splenic immune cell composition was analyzed.

### Preparation of human tonsillar naive CD4^+^ T cells

Human tonsil samples were obtained from children aged 3–12 years at the Department of Otolaryngology-Head and Neck Surgery of Seoul St. Mary’s Hospital. The experimental procedures were approved by the Institutional Review Board (IRB) of the Catholic University of Korea (KC18TESI0723). Written informed consent for sample collection and research use was obtained by the hospital from the participants or their parents/legal guardians. All procedures involving human samples were conducted in accordance with the principles of the Declaration of Helsinki and the Department of Health and Human Services Belmont Report. The tonsil tissue was cut into small pieces and homogenized by passing through a 70 µm cell strainer using a plastic syringe plunger. The samples were resuspended in cold 1× HBSS (Thermo Fisher Scientific) supplemented with 10% fetal bovine serum (FBS) and 1% penicillin/streptomycin. Mononuclear cells (MNCs) were isolated via density gradient centrifugation using Lymphoprep (Stem Cell Technologies). After washing with PBS supplemented with 1.5% FBS, human naive CD4^+^ T cells were isolated from MNCs using a human naive CD4^+^ T cell isolation kit II (Miltenyi Biotec).

### Flow cytometry

Flow cytometry was performed as previously described (Park and Lee, 2025; Song and Lee, 2024), with minor modifications. Immune cells were isolated directly from the spleen or after in vitro stimulation of splenocytes. For surface staining, cells were incubated for 30 min at 4 °C in PBS containing 1.5% FBS with the following fluorochrome-conjugated monoclonal antibodies, each diluted 1:200: anti-Thy1.1-PerCP-Cy5.5 (HIS51, eBioscience), anti-CD4-PE (RM4-5, BioLegend), anti-CD4-PerCP-Cy5.5 (RM4-5, Tonbo Biosciences), anti-PD-1-FITC (J43, eBioscience), anti-CD8a-PE (53-6.7, Tonbo Biosciences), anti-CD44-FITC (IM7, BD Biosciences), anti-CD62L-PE-Cy7 (MEL-14, BD Biosciences), anti-CD25-PE (PC61, BioLegend), anti-ICOS-APC (7E.17G9, BioLegend), anti-CD19-Bv421 (1D3, BD Biosciences), anti-CD19-APC (1D3/CD19, BioLegend), anti-GL7-AlexaFluor488 (GL7, BioLegend), anti-GL7-PE (GL7, BioLegend), and anti-CD138-Bv421 (281-2, BioLegend). Ghost dye Violet 510 (Tonbo Biosciences) was diluted 1:1000 in PBS supplemented with 1.5% FBS. For CXCR5 and FAS staining, a two-step staining procedure was performed. Cells were incubated for 30 min with biotinylated anti-CXCR5 (1:200 dilution, 2G8, BD Biosciences) or anti-CD95 (1:200 dilution, Jo2, BD Biosciences) antibodies. This was followed by a 30-min incubation with streptavidin-PE-Cy7 (1:200 dilution, BioLegend). For intracellular staining, cells were fixed and permeabilized with the Foxp3/Transcription factor staining buffer set (eBioscience) in accordance with the manufacturer’s protocol, and then stained with the following antibodies, each diluted 1:100: anti-Bcl-6-AlexaFluor647 (K112-91, BD Biosciences), anti-FOXP3-eFlour450 (FJK-16s, eBioscience), anti-T-bet-PE (4B10, eBioscience), anti-GATA3-PE-Cy7 (TWAJ, eBioscience), anti-Rorγt-APC (B2D, eBioscience), anti-Blimp1-PE (5E7, BioLegend), and anti-Ki-67-APC (SolA15, eBioscience). For intracellular cytokine staining, cells were stimulated with phorbol 12-myristate 13-acetate (PMA) and ionomycin in the presence of GolgiStop and GolgiPlug (BD Biosciences) for 4 h, followed by staining with the following antibodies, each diluted 1:100: anti-IFN-γ-APC (XMG1.2, BioLegend), anti-IL-4-PE-Cy7 (11B11, BD Biosciences), and anti-IL-17A-PE (TC11-18H10, BD Biosciences). Cell suspensions obtained after in vitro stimulation of human tonsillar cells were stained with the following fluorescent dye-conjugated antibodies, each diluted 1:100: anti-CD4-FITC (OKT4, BioLegend), anti-CXCR5-By421 (RF8B2, BD Biosciences), anti-PD-1-PE-Cy7 (EH12.2H7, BioLegend), and anti-CD45RA-PerCP-Cy5.5 (HI100, BioLegend). All the stained cells were analyzed on a FACSymphony A5 cell analyzer (BD Biosciences) or a CytoFLEX LX flow cytometer (Beckman Coulter) at the Microbiome Core Facility of POSTECH. Flow cytometry data were processed using the FlowJo software (BD Biosciences).

### In vitro T helper-cell differentiation assay

The differentiation of T helper cells was assayed using a previously described method (Park et al, 2024). Naive CD4^+^ T cells were isolated from spleen and lymph nodes via negative selection using the EasySep™ Mouse Streptavidin RapidSphere^TM^ isolation kit (Stem Cell Technologies) in combination with biotinylated antibodies against CD19 (6D5, BioLegend), B220 (RA3-6B2, BioLegend), CD8a (53-6.7, BioLegend), CD11b (M1/70, BioLegend), CD11c (N418, BioLegend), CD49b (DX5, BioLegend), Gr-1 (RB6-8C5, BioLegend), TER-119 (TER-119, BioLegend), TCR gamma/delta (UC7-13D5, eBioscience), and CD44 (IM7, BioLegend). The cells were stimulated with plate-bound anti-CD3 (145-2C11, BD Pharmingen) (1 μg/mL) and anti-CD28 (37.51, BD Pharmingen) (1 μg/mL) under the following differentiation conditions: T_H_0 differentiation [anti-IL-4 (11B11, BioXcell) (10 μg/mL) and anti-IFN- γ (XMG1.2, BioXcell) (10 μg/mL)]; T_H_1 differentiation [IL-12 (R&D Systems) (10 ng/mL) and anti-IL-4 (10 μg/mL)]; T_H_2 differentiation [IL-4 (Peprotech) (10 ng/mL) and anti-IFN- γ (10 μg/mL)]; T_H_17 differentiation [IL-6 (Peprotech) (30 ng/mL), IL-1β (Peprotech) (20 ng/mL), TGF-β (BioLegend) (2 ng/mL), anti-IL-4 (10 μg/mL) and anti-IFN-γ (10 μg/mL)]; and T_REG_ differentiation [IL-2 (Peprotech) (10 ng/mL) and TGF-β (2 ng/mL)]. For each condition, OCA (50 μM) or vehicle (DMSO) was added. After culture, the cells were analyzed on a FACSymphony A5 flow cytometer (BD Biosciences), and data were processed using the FlowJo software (BD Biosciences).

### In vitro differentiation of T_FH_-like cells

The differentiation of T_FH_-like cells was assayed using a previously described method (Park et al, 2024). Naive CD4^+^ T cells were isolated from the spleen of *Etv5*^*f/f*^ and *Etv5*^*f/f*^*;Cd4-Cre* mice and stimulated with plate-bound anti-CD3 (1 μg/mL) and anti-CD28 (1 μg/mL). Cells were cultured for 3 days under T_H_0 conditions (anti-IL-4, 10 μg/mL; anti-IFN-γ, 10 μg/mL) or T_FH_-polarizing conditions [IL-6 (Peprotech), 100 ng/mL; IL-21 (Peprotech), 50 ng/mL; anti-IL-4, 10 μg/mL; anti-IFN-γ, 10 μg/mL; anti-TGF-β, 10 μg/mL] in the presence of OCA (50 μM) or vehicle control (DMSO). The cells were harvested and analyzed via RT-qPCR, ChIP–qPCR, and ELISA.

### In vitro mouse T_FH_ cell differentiation using OT-II cells

The experiment was performed as previously described (Gao et al, 2020; Park et al, 2024). Briefly, splenocytes from Thy1.2^+^ C57BL/6 mice were stimulated with lipopolysaccharide (LPS) (Sigma-Aldrich) (1 μg/mL) for 24 h and cocultured with Thy1.1^+^ naive OT-II cells isolated from OT-II; *Etv5*^*f/f*^ and OT-II; *Etv5*^*f/f*^*;Cd4-Cre* mice at a 10:1 ratio in the presence of ovalbumin peptide 323–339 (InvivoGen, 1 μg/mL), IL-6 (100 ng/mL), and IL-21 (50 ng/mL) for 3 days with OCA (50 μM) or vehicle (DMSO). For apoptosis assay, cells were harvested and stained using the APC Annexin V Apoptosis Detection Kit with propidium iodide (PI) (BioLegend) according to the manufacturer’s protocol. The stained cells were analyzed via flow cytometry.

### In vitro human T_FH_ cell differentiation

The experiment was performed using a previously described method (Kim et al, 2018; Locci et al, 2016; Park et al, 2024). Human naive CD4^+^ T cells were purified from tonsils and seeded into 96-well U-bottom plates. The cells were cultured for 3 days with IL-12 (Humanzyme, 5 ng/mL), TGF-β (BioLegend, 1 ng/mL), and Dynabeads™ human T-Activator CD3/CD28 (Thermo Fisher Scientific) at a bead-to-cell ratio of 1:1. For T_H_0 conditions, cells were stimulated with Dynabeads™ Human T-Activator CD3/CD28 alone. OCA (50 μM) or vehicle (DMSO) was added at the initiation of culture (day 0). After 3 days, the cells were collected and analyzed via RT-qPCR and flow cytometry.

### Western blotting

Naive CD4^+^ T cells isolated from the spleen were cultured under T_FH_-polarizing conditions in the presence of OCA (50 μM) or vehicle control (DMSO). The cells were lysed in RIPA buffer (50 mM Tris-HCl pH 7.4, 150 mM NaCl, 1 mM PMSF, 1% NP-40, 0.5% sodium deoxycholate, 0.1% SDS, 2× Complete Protease Inhibitor Cocktail (5892953001, Roche) containing 10× PhosSTOP™ (4906837001, Roche). Protein samples were separated via SDS-PAGE and then transferred onto 0.45 μm nitrocellulose membranes (Bio-Rad). The membranes were incubated overnight at 4 °C with the following primary antibodies: anti-ETV5 (1:1000 dilution, 13011-1-AP, Proteintech), and anti-α-tubulin (1:1000 dilution, sc-398103, Santa Cruz Technology). Thereafter, HRP-conjugated secondary antibodies (1:3000 dilution) were added and the membranes were further incubated for 1 h at room temperature (RT). Proteins were visualized using Immobilon Western Chemiluminescent HRP Substrate (Merck Millipore). Western blots were imaged using an ImageQuant LAS 500 (GE Healthcare Life Sciences).

### OVA immunization and OCA treatment

*Etv5*^*f/f*^ and *Etv5*^*f/f*^*;Cd4*-*Cre* mice were intravenously administered OCA (2.5 mg/kg) or vehicle (10% DMSO in PBS). The next day, mice received an intraperitoneal injection of ovalbumin (OVA; 50 μg, InvivoGen) emulsified in 200 μL alum (InvivoGen). Three days after immunization, a second intravenous dose of OCA (2.5 mg/kg) or vehicle was administered. Four days following the second injection, populations of splenic immune cells were analyzed via flow cytometry.

For the OPN rescue experiment, mice were intravenously administered OCA (2.5 mg/kg) or vehicle (10% DMSO in PBS) on days 0 and 4. Recombinant mouse osteopontin (OPN; 3 μg; 441-OP-050, R&D Systems) or PBS was intravenously injected on days 1 and 5. Two hours after the first OPN or PBS injection on day 1, mice were intraperitoneally immunized with 50 μg OVA in 200 μL alum. Mice were sacrificed on day 8, and immune cell frequencies were analyzed via flow cytometry.

### Immunofluorescence staining

The kidneys were dissected, embedded in Tissue-Tek OCT compound (Sakura), snap-frozen, and sectioned at a thickness of 8 μm using a cryostat microtome (Leica). The sections were fixed in 10% formalin (Sigma-Aldrich) and washed with PBS. For Fc blocking, the sections were incubated with anti-CD16/CD32 antibodies (1:200 dilution, 2.4G2, eBioscience) diluted in PBS containing 0.05% Triton X-100 (Sigma-Aldrich) for 30 min at RT. After washing with PBS, the kidney sections were stained with anti-IgG-FITC antibody (1:100 dilution, F0257, Sigma-Aldrich) for 2 h in a humid chamber. Nuclei were subsequently counterstained with DAPI (1:1500 dilution, D9542, Sigma-Aldrich) for 1 min at RT. The sections were washed with PBS and mounted with mounting medium (Dako). Images were acquired at 10× magnification using an Olympus CKX53 Inverted Phase Contrast Microscope.

### Tissue histology

Dissected liver, lung, and kidney tissues were fixed overnight in 10% formalin at 4 °C. For paraffin embedding, the fixed tissues were dehydrated through a graded ethanol series (70%, 80%, 90%, and 100%) and cleared with xylene. The tissues were then embedded in paraffin and sectioned at a thickness of 4 µm using a semi-automated rotary microtome (RM2245, Leica Biosystems). The sections were mounted on adhesive slides (Duran). They were deparaffinized and rehydrated through a graded series of xylene, 100% ethanol, 95% ethanol, 85% ethanol, and 70% ethanol, and then washed with distilled water. Hematoxylin (Sigma-Aldrich) and eosin (Cancer Diagnostics) staining was performed, and sections were dehydrated by passing through reverse graded ethanol and xylene before mounting with Canada balsam (Sigma-Aldrich). Images were acquired at ×10 magnification using a BX43 microscope (Olympus).

### Enzyme-linked immunosorbent assay (ELISA)

Corning 96-well half area clear flat bottom microplates (Sigma-Aldrich) were precoated overnight with 2 μg/mL anti-mouse Ig (Southern Biotechnology) at 4 °C. The following day, the plates were washed with PBS containing 0.05% Tween-20 (PBST) and blocked with 0.5% bovine serum albumin (BSA) for 1 h at RT. Diluted serum samples and mouse reference serum (Bethyl) were added (50 μL/well) and the plates were incubated for 2 h at RT. After washing with PBST, the plates were incubated with horseradish peroxidase (HRP)-conjugated secondary antibodies for 1 h at RT. Goat anti-mouse IgG (H + L), IgG1, IgG2c, and IgM (Southern Biotechnology) were used to quantify total IgG, IgG1, IgG2c, and IgM, respectively. 50 μL of TMB substrate (Squamodisc) was added to the plates and allowed to stand for 5 min in the dark; the reaction was stopped with 50 μL of 1 M H_2_SO_4_. Absorbance was measured at 450 nm, and serum immunoglobulin concentrations were calculated using standard curves. For anti-dsDNA antibody detection, plates were precoated overnight with 200 μg/mL calf thymus DNA (Invitrogen) at 4 °C, and the remainder of the assay was performed as described above. For anti-OVA antibody detection, plates were precoated overnight with 50 μg/mL OVA at 4 °C, and the remainder of the assay was performed as described above. OPN levels in serum were measured using the DuoSet mouse osteopontin ELISA kit (DY441, R&D Systems) according to the manufacturer’s instructions. For in vitro assays, cells were cultured for 72 h under T_FH_-like cell differentiation conditions in the presence of OCA or vehicle (DMSO). The supernatants were collected and assayed for OPN levels using the DuoSet mouse osteopontin ELISA kit (DY441, R&D Systems) according to the manufacturer’s instructions.

### Chromatin immunoprecipitation–quantitative polymerase chain reaction (ChIP–qPCR)

ChIP–qPCR was performed as previously described (Hong et al, 2022; Lee et al, 2025; Park et al, 2024; Song et al, 2025). Naive CD4^+^ T cells isolated from the spleen of *Etv5*^*f/f*^ and *Etv5*^*f/f*^*;Cd4*-*Cre* mice were cultured under T_FH_-polarizing conditions (IL-6, 100 ng/mL; IL-21, 50 ng/mL; anti-IL-4, 10 μg/mL; anti-IFN-γ, 10 μg/mL; anti-TGF-β, 10 μg/mL) for 3 days in the presence of OCA or vehicle (DMSO). The cultured cells were crosslinked with 1% paraformaldehyde for 10 min at RT with constant agitation, and the reaction was stopped with 1 M glycine. After 5 min, the cells were washed twice with cold PBS. The cells were lysed in buffer 1 (50 mM HEPES-KOH pH 7.5, 140 mM NaCl, 1 mM EDTA pH 8.0, 10% glycerol, and 0.5% NP-40) for 10 min at 4 °C, and the cells were then resuspended in buffer 2 (10 mM Tris-HCl, pH 8.0; 300 mM NaCl; 0.1% sodium deoxycholate; 1% Triton X-100; 1 mM EDTA; 0.5 mM EGTA) and sonicated. After centrifugation at 9400 rpm for 10 min, the supernatant was precleared with protein G agarose (Millipore) for 1 h at 4 °C. After the preclearing step, 3 μg normal rabbit IgG (Cell Signaling Technology) or anti-ETV5 antibody (13011-1-AP, ProteinTech) was allowed to bind overnight incubation at 4 °C with gentle rotation. The next day, the chromatin and antibody mixtures were further incubated with protein G agarose for 4 h at 4 °C. After washing with low salt buffer (2 mM EDTA, 1% Triton X-100, 20 mM Tris-HCl pH 8.0, 150 mM NaCl, and 0.1% SDS), high salt buffer (2 mM EDTA, 1% Triton X-100, 20 mM Tris-HCl pH 8.0, 500 mM NaCl, and 0.1% SDS), LiCl buffer (0.25 M LiCl, 10 mM Tris-HCl pH 8.0, 1 mM EDTA, 1% NP-40, and 1% sodium deoxycholate), and TE buffer (10 mM Tris-HCl pH 8.0 and 1 mM EDTA), the bound chromatin was eluted twice with the elution buffer (0.1 M NaHCO_3_ and 0.5% SDS) and reverse-crosslinked overnight at 65 °C in 200 mM NaCl. Proteins were digested with protease K. DNA was purified using Expin CleanUp SV (GeneAll). Finally, quantitative PCR (qPCR) was performed to quantify the relative enrichment of *Spp1* promoter regions in the immunoprecipitated DNA fragments.

### RNA extraction and real-time quantitative polymerase chain reaction (RT-qPCR)

RT-qPCR was performed as previously described (Bong et al, 2024; Kim et al, 2025), with minor modifications. Total RNA was purified from the cells using RiboEx (GeneAll) according to the manufacturer’s instructions. cDNA was synthesized with the GoScript™ Reverse Transcription System (Promega) according to the manufacturer’s instructions. qPCR was performed using SYBR Green Master Mix (Toyobo), and gene expression levels were normalized to those of *Hprt*.

### Statistical analysis

Statistical analyses were performed using Prism 10.5.0 (GraphPad Software). Data are presented as mean ± standard error of the mean (SEM). Statistical significance was determined using two-tailed unpaired Student’s *t* tests for prespecified pairwise comparisons, as indicated in the figure legends. For experiments with three or more groups, only comparisons of interest were tested, typically between the control and each indicated experimental group. *P* values < 0.05 were considered to indicate statistically significant differences. Sample sizes and the numbers of independent experiments are indicated in the corresponding figure legends.

### Graphics

Visual abstract and schematic illustrations for experimental design in Figs. 1, 2, 3, 4 were created with BioRender.com.

## Supplementary information

Appendix

Peer Review File

Source data Fig. 1

Source data Fig. 2

Source data Fig. 3

Source data Fig. 4

Figure EV1 Source Data

Figure EV2 Source Data

Figure EV3 Source Data

Figure EV4 Source Data

Figure EV5 Source Data

Expanded View Figures

## Data Availability

The source data of this paper are collected in the following database record: biostudies: S-BIAD3311 (10.6019/S-BIAD3311). The source data of this paper are collected in the following database record: biostudies:S-SCDT-10_1038-S44321-026-00478-6.
