# Supplementary material for: Obeticholic acid alleviates lupus pathology by inhibiting T follicular helper cell differentiation
Source: EMBO Mol Med. 2026 Jul 3;18(8):3258–77. doi: 10.1038/s44321-026-00478-6 (PMC13469628; doi:10.1038/s44321-026-00478-6)
Supplement: Supplementary file 1 [file 44321_2026_478_MOESM12_ESM.pdf]

Expanded View Figures

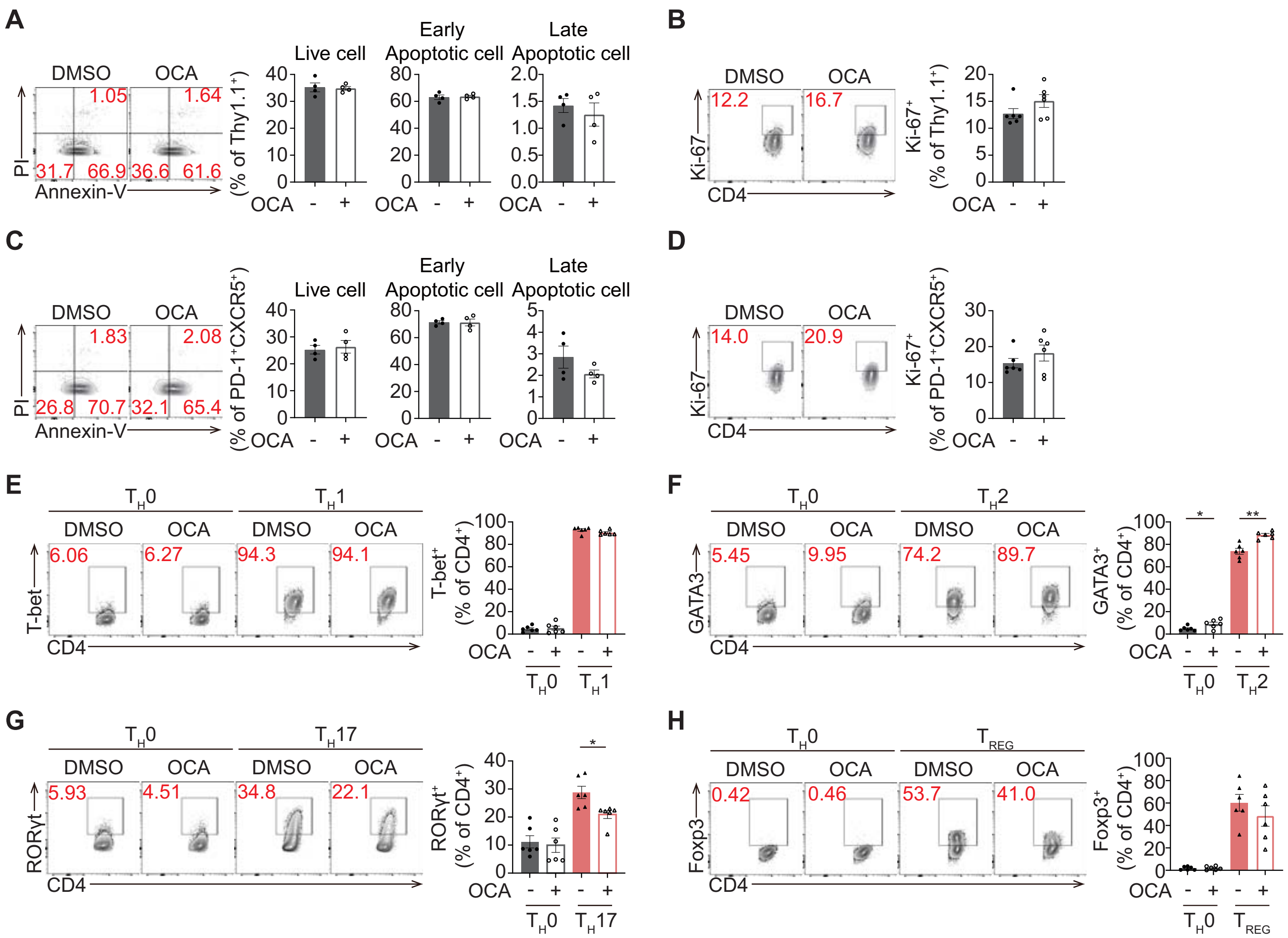

Figure EV1. Effect of obeticholic acid (OCA) treatment on survival, proliferation, and differentiation of CD4⁺ T cells in vitro.

(**A**) Flow cytometric analysis of apoptosis in Thy1.1⁺ WT OT-II cells cultured under T follicular helper (T_{FH})-polarizing conditions in the presence of dimethyl sulfoxide (DMSO) or OCA. Frequencies of live (PI^-Annexin V^-), early apoptotic (PI^-Annexin V^+), and late apoptotic (PI^+Annexin V^+) cells were quantified ($n = 4$ per group). Data are representative of two independent experiments. (**B**) Flow cytometric analysis of Ki-67⁺ Thy1.1⁺ WT OT-II cells after 3 days of T_{FH}-polarizing culture in the presence of DMSO or OCA ($n = 6$ per group). Data are representative of three independent experiments. (**C**) Flow cytometric analysis of apoptosis in CXCR5⁺PD-1⁺ WT OT-II cells cultured under T_{FH}-polarizing conditions in the presence of DMSO or OCA. Frequencies of live, early apoptotic, and late apoptotic cells were quantified ($n = 4$ per group). Data are representative of two independent experiments. (**D**) Flow cytometric analysis of Ki-67⁺ CXCR5⁺PD-1⁺ WT OT-II cells after 3 days of T_{FH}-polarizing culture in the presence of DMSO or OCA ($n = 6$ per group). Data are representative of three independent experiments. (**E–H**) In vitro T helper-cell differentiation assays. Naive CD4⁺ T cells were cultured under T_H1- (**E**), T_H2- (**F**), T_H17- (**G**), or T_{REG} (**H**)-polarizing conditions, in the presence of DMSO or OCA. Data are representative of two independent experiments. Bar graphs present the data as mean ± SEM values. Statistical significance was determined using two-tailed unpaired Student's *t* tests for the indicated pairwise comparisons. Each dot represents a biological replicate. *$P < 0.05$ and **$P < 0.01$. Exact *P* values are in Appendix Table S1. Source data are available online for this figure.

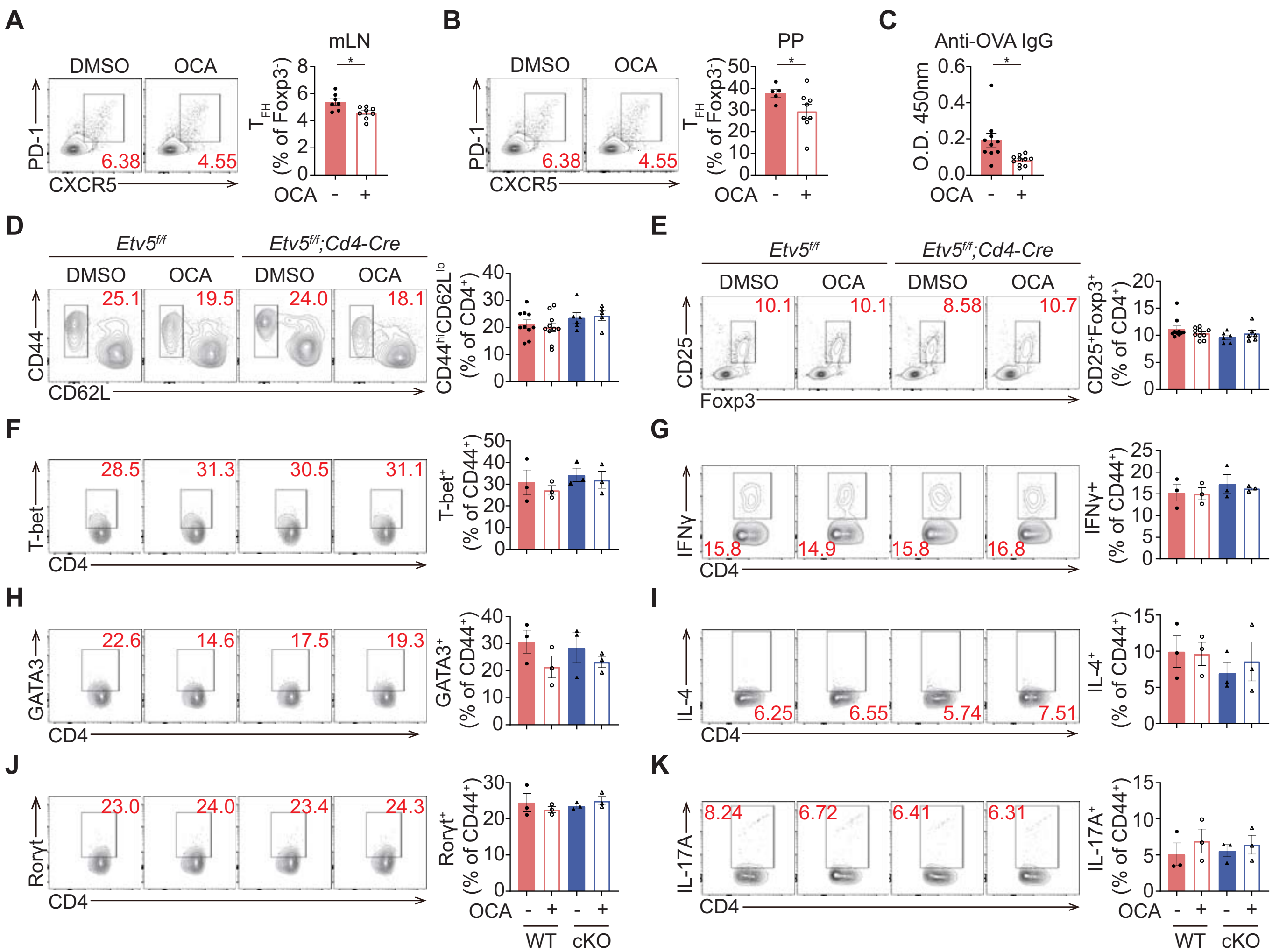

Figure EV2. Effect of obeticholic acid (OCA) treatment on T_{FH} and CD4⁺ T cell subsets after ovalbumin (OVA) immunization.

(**A**) Flow cytometric analysis of CXCR5⁺PD-1⁺ T_{FH} cells in the mesenteric lymph nodes (mLN) from mice immunized with OVA in alum and treated with dimethyl sulfoxide (DMSO; $n = 7$) or OCA ($n = 8$). Data are representative of two independent experiments. (**B**) Flow cytometric analysis of CXCR5⁺PD-1⁺ T_{FH} cells in the Peyer's patches (PP) from mice immunized with OVA in alum and treated with DMSO ($n = 5$) or OCA ($n = 8$). Data are representative of two independent experiments. (**C**) ELISA of serum anti-OVA IgG levels in mice immunized with OVA in alum and treated with DMSO or OCA ($n = 10$ per group). Data are representative of two independent experiments. (**D**, **E**) Flow cytometric analysis of CD4⁺ T cell subsets in OVA-immunized *Etv5^f/f^* (WT) and *Etv5^f/f^;Cd4-Cre* (cKO) mice treated with DMSO or OCA. Shown are the frequencies of effector CD4⁺ T cells (CD44^hi^CD62L^lo^) (**D**), T_{REG} cells (CD25⁺Foxp3⁺) (**E**) from WT ($n = 10$) and cKO ($n = 6$) mice. Data are representative of three independent experiments. (**F–K**) Flow cytometric analysis of CD4⁺ T cell subsets in OVA-immunized *Etv5^f/f^* (WT) and *Etv5^f/f^;Cd4-Cre* (cKO) mice treated with DMSO or OCA. Shown are the frequencies of T_H1 cells (T-bet⁺ or IFN-γ⁺) (**F**, **G**), T_H2 cells (GATA3⁺ or IL-4⁺) (**H**, **I**), and T_H17 cells (Rorγt⁺ or IL-17a⁺) (**J**, **K**) ($n = 3$ per group). Data are from a single experiment. Bar graphs present the data as mean ± SEM values. Statistical significance was determined using two-tailed unpaired Student's *t* tests for the indicated pairwise comparisons. Each dot represents an individual mouse. $*P < 0.05$. Exact *P* values are provided in Appendix Table S1. Source data are available online for this figure.

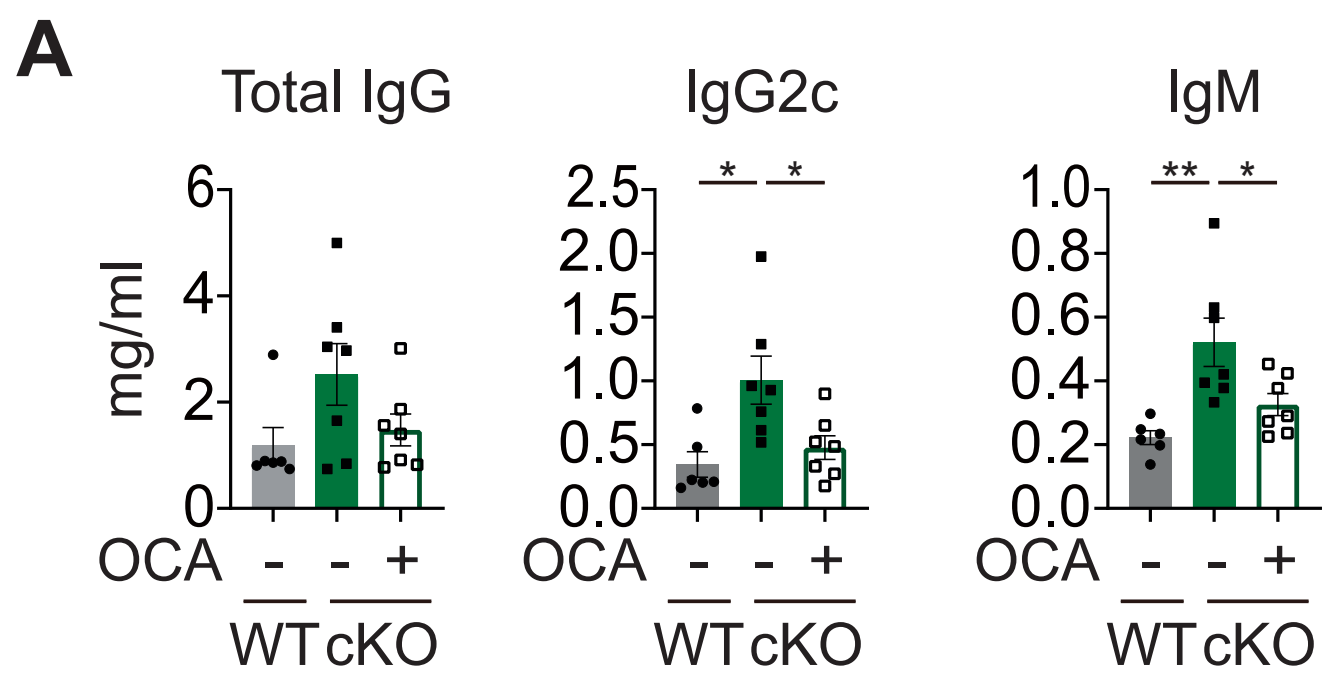

Figure EV3. Effect of obeticholic acid (OCA) treatment on serum immunoglobulin levels in T-cell-specific CIC-deficient mice.

(**A**) ELISA of serum levels of total IgG, IgG2c, and IgM in *Cic*$^{f/f}$ (wild-type; WT) and *Cic*$^{f/f}$*;Cd4-Cre* (cKO) mice treated with dimethyl sulfoxide (DMSO) or OCA (WT, $n = 6$ and cKO, $n = 7$). Data are representative of three independent experiments. Bar graphs present the data as mean ± SEM values. Statistical significance was determined using two-tailed unpaired Student's t tests for the indicated pairwise comparisons. Each dot represents an individual mouse. *$P < 0.05$ and **$P < 0.01$. Exact P values are provided in Appendix Table S1. Source data are available online for this figure.

 © The Author(s)

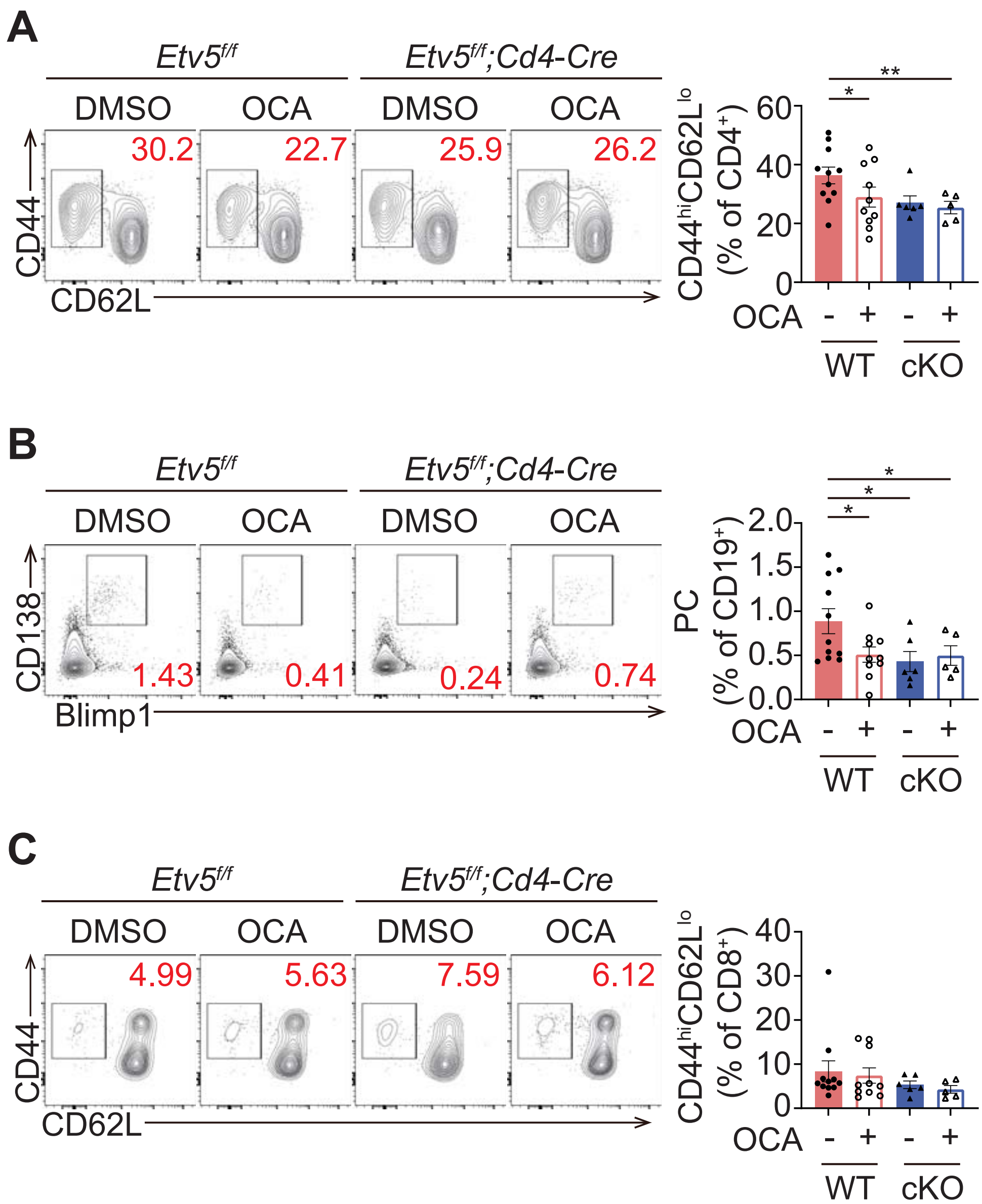

Figure EV4. Effects of obeticholic acid (OCA) on effector T-cell and plasma-cell differentiation in pristane-induced lupus mouse model.

(**A–C**) Flow cytometric analysis of T cells and plasma cells in *Etv5*$^{f/f}$ (wild-type; WT) and *Etv5*$^{f/f}$*;Cd4-Cre* (cKO) mice treated with pristane and subsequently administered dimethyl sulfoxide (DMSO) or OCA. Shown are the frequencies of CD4$^+$ effector T cells (CD44^{hi}CD62L^{lo}) (**A**), CD138$^+$Blimp1$^+$ plasma cells (**B**), and CD8$^+$ effector T cells (CD44^{hi}CD62L^{lo}) (**C**) (WT DMSO, $n = 11$; WT OCA, $n = 10$; cKO DMSO, $n = 6$; cKO OCA, $n = 5$). Data are representative of five independent experiments. Bar graphs present the data as mean ± SEM values. Statistical significance was determined using two-tailed unpaired Student's *t* tests for the indicated pairwise comparisons. Each dot represents an individual mouse. *$P < 0.05$ and **$P < 0.01$. Exact *P* values are provided in Appendix Table S1. Source data are available online for this figure.

© The Author(s)

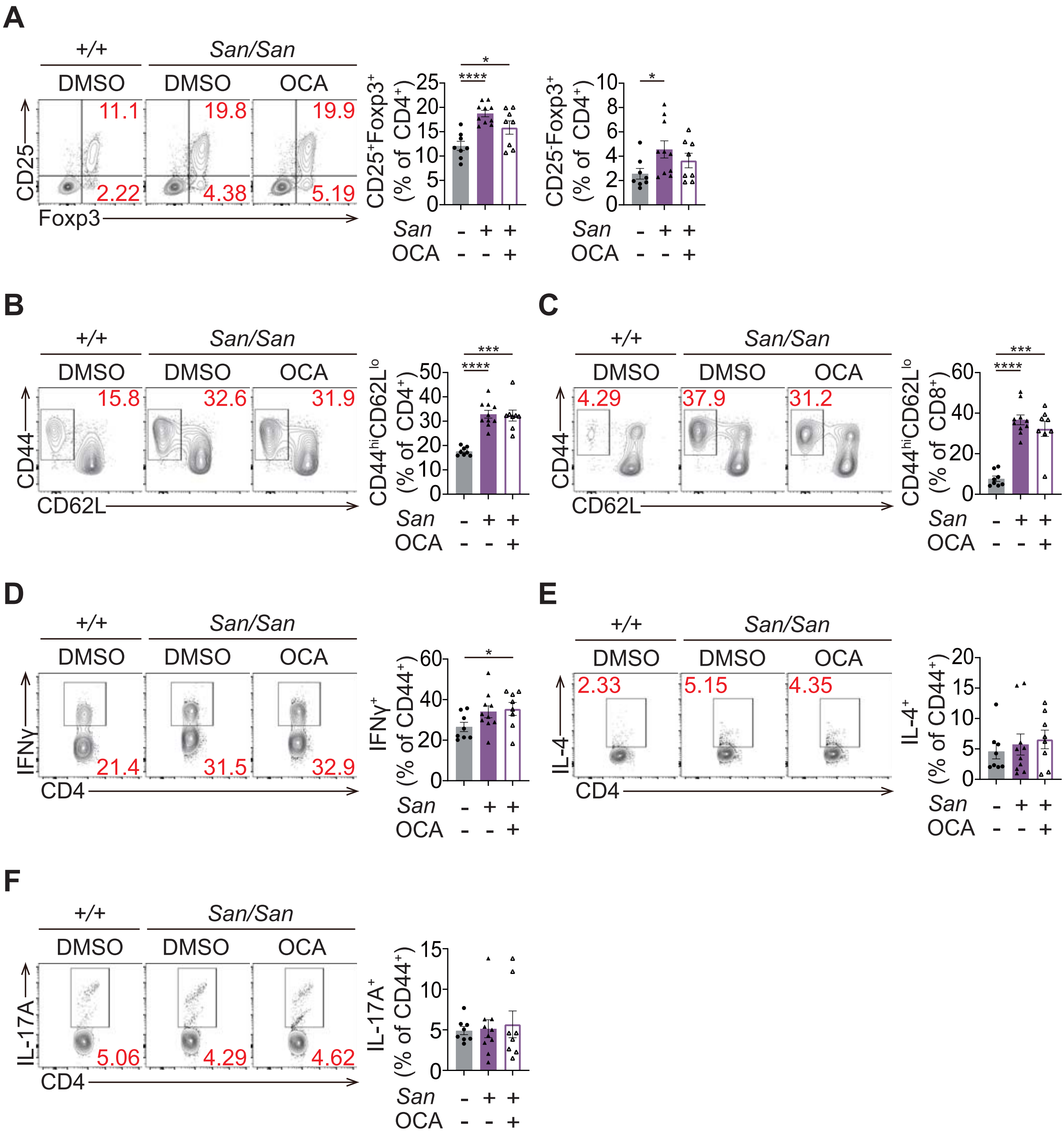

Figure EV5. Effects of obeticholic acid (OCA) on T-cell activation and differentiation in *Sanroque* mice.

(A–F) Flow cytometric analysis of T-cell subsets in wild-type (WT) and *Sanroque* mice treated with dimethyl sulfoxide (DMSO) or OCA. Shown are the frequencies of T_{REG} cells ($CD25^+Foxp3^+$ and $CD25^-Foxp3^+$) (**A**), $CD4^+$ effector T cells ($CD44^{hi}CD62L^{lo}$) (**B**), $CD8^+$ effector T cells ($CD44^{hi}CD62L^{lo}$) (**C**), T_H1 cells (IFN-γ^+) (**D**), T_H2 cells (IL-4^+) (**E**), and T_H17 cells (IL-$17a^+$) (**F**). WT mice were treated with DMSO ($n = 8$) and Sanroque mice were treated with either DMSO ($n = 10$) or OCA ($n = 8$). Data are representative of five independent experiments. Bar graphs present the data as mean ± SEM values. Statistical significance was determined using two-tailed unpaired Student's *t* tests for the indicated pairwise comparisons. Each dot represents an individual mouse. $^*P < 0.05$, $^{***}P < 0.001$, and $^{****}P < 0.0001$. Exact *P* values are provided in Appendix Table S1. Source data are available online for this figure.

© The Author(s)
