# Supplementary material for: Obeticholic acid alleviates lupus pathology by inhibiting T follicular helper cell differentiation
Source: EMBO Mol Med. 2026 Jul 3;18(8):3258–77. doi: 10.1038/s44321-026-00478-6 (PMC13469628; doi:10.1038/s44321-026-00478-6)
Supplement: Supplementary file 2 [file 44321_2026_478_MOESM1_ESM.pdf]

Appendix for “Obeticholic acid alleviates lupus pathology by inhibiting T follicular helper cell differentiation”

Table of Contents

1. Appendix Figure S1 .... **2**

2. Appendix Figure S2 .... **3**

3. Appendix Table S1 .... **4**

Appendix Figure S1

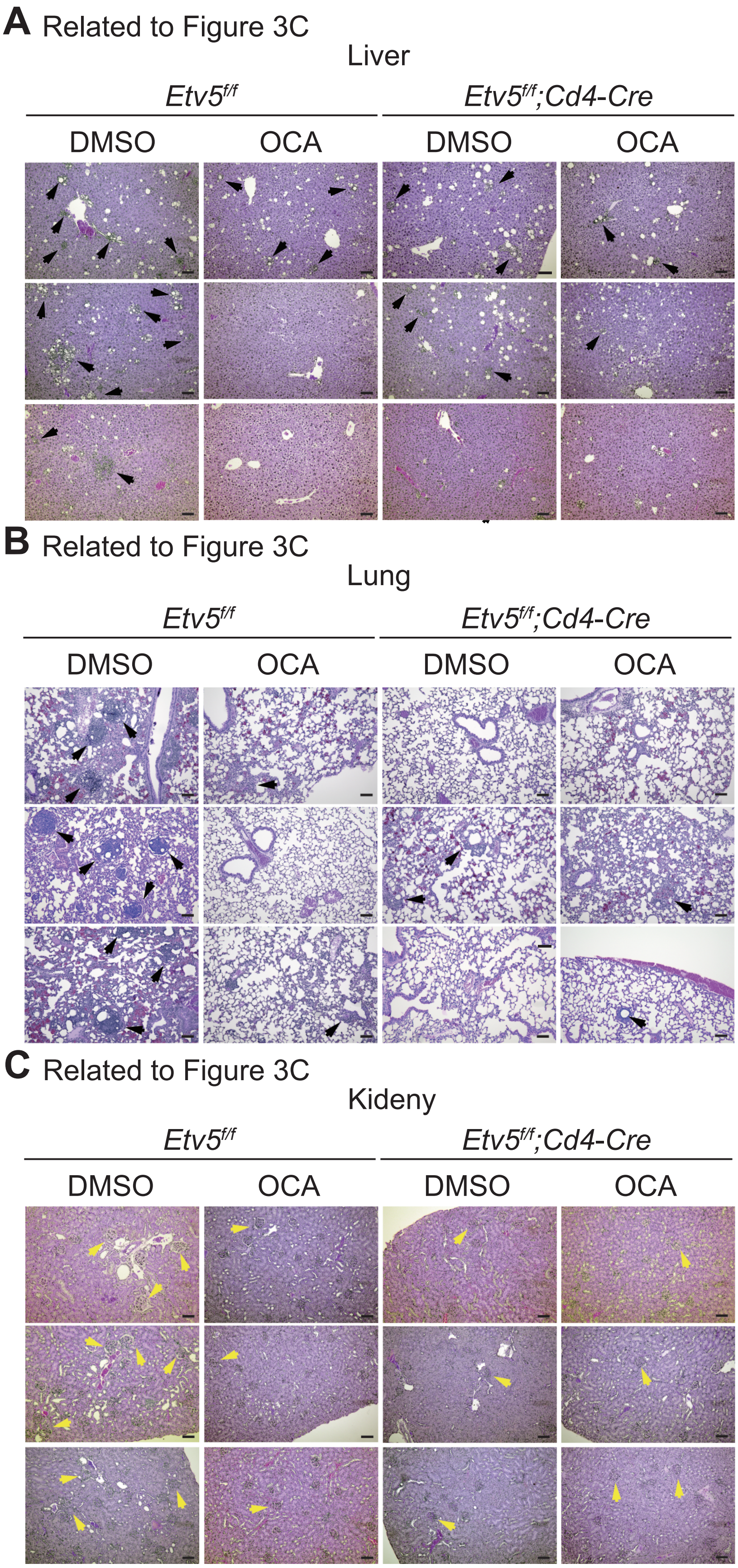

Appendix Figure S1. Additional hematoxylin and eosin (H&E) images supporting the representative histological features of pristane-induced lupus mouse models.

(A-C) Additional H&E staining of the liver (A), lung (B), and kidney (C) sections from Etv5f/f (wild-type; WT) and Etv5f/f;Cd4-Cre (cKO) mice treated with dimethyl sulfoxide (DMSO) or obeticholic acid (OCA), corresponding to the representative images shown in Figure 3C. Three representative images are shown for each group. Black arrows indicate immune cell infiltration; yellow arrows indicate glomerular abnormalities. Scale bar, 100 µm.

Appendix Figure S2

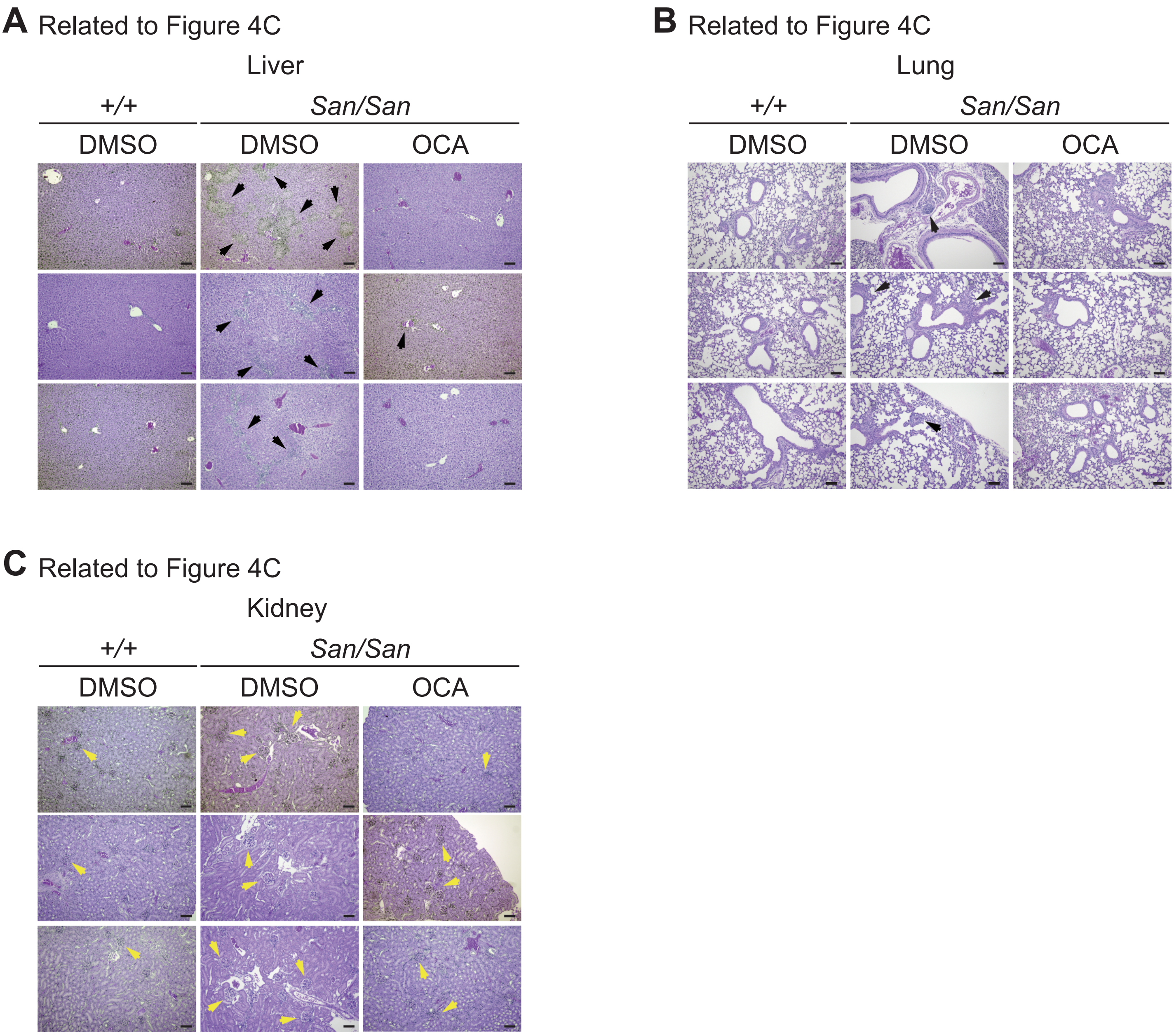

Appendix Figure S2 – Additional hematoxylin and eosin (H&E) images supporting the representative histological features of Sanroque mice.

(A-C) Additional H&E staining of the liver (A), lung (B), and kidney (C) sections from wild-type (WT) and Sanroque mice treated with dimethyl sulfoxide (DMSO) or obeticholic acid (OCA), corresponding to the representative images shown in Figure 4C. Three representative images are shown for each group. Black arrows indicate immune cell infiltration; yellow arrows indicate glomerular abnormalities. Scale bar, 100 µm.

Appendix Table S1. Exact P value.

Figure	Groups	Exact P value
Figure 1A	WT_DMSO in PD-1$^+$CXCR5$^+$ vs WT_OCA in PD-1$^+$CXCR5$^+$	0.0167
Figure 1A	WT_DMSO in PD-1$^+$CXCR5$^+$ vs cKO_DMSO in PD-1$^+$CXCR5$^+$	0.0384
Figure 1A	WT_DMSO in PD-1$^+$CXCR5$^+$ vs cKO_OCA in PD-1$^+$CXCR5$^+$	0.0182
Figure 1A	WT_DMSO in BCL6$^+$ vs WT_OCA in BCL6$^+$	0.0005
Figure 1A	WT_DMSO in BCL6$^+$ vs cKO_DMSO in BCL6$^+$	0.0012
Figure 1A	WT_DMSO in BCL6$^+$ vs cKO_OCA in BCL6$^+$	<0.0001
Figure 1B	WT_DMSO vs WT_OCA	0.0003
Figure 1B	WT_DMSO vs cKO_DMSO	0.0029
Figure 1B	WT_DMSO vs cKO_OCA	0.0008
Figure 1D	WT_DMSO vs WT_OCA	0.0013
Figure 1D	WT_DMSO vs cKO_DMSO	0.0027
Figure 1D	WT_DMSO vs cKO_OCA	<0.0001
Figure 1F	WT_DMSO vs WT_OCA	0.0017
Figure 1F	WT_DMSO vs cKO_DMSO	0.0241
Figure 1F	WT_DMSO vs cKO_OCA	0.0057
Figure 1G	WT_DMSO vs WT_OCA	0.0015
Figure 1G	WT_DMSO vs cKO_DMSO	0.0409
Figure 1G	WT_DMSO vs cKO_OCA	<0.0001
Figure 1H	WT_DMSO vs cKO_DMSO	0.0001
Figure 1H	WT_DMSO vs cKO_OCA	0.0002
Figure 1H	cKO_DMSO vs cKO_OCA	0.0488
Figure 1I	WT_DMSO vs cKO_DMSO	<0.0001

Figure 1I	WT_DMSO vs cKO_OCA	<0.0001
Figure 1I	cKO_DMSO vs cKO_OCA	0.0179
Figure 1J	WT_DMSO vs cKO_DMSO	<0.0001
Figure 1J	WT_DMSO vs cKO_OCA	0.0005
Figure 1J	cKO_DMSO vs cKO_OCA	0.0440
Figure 2A	$T_{H}0$_DMSO vs $T_{H}0$_OCA	0.0034
Figure 2A	T_{FH}_DMSO vs T_{FH}_OCA	0.0364
Figure 2B	DMSO vs OCA	<0.0001
Figure 2C	Rabbit IgG_DMSO vs ETV5_DMSO	0.0280
Figure 2C	ETV5_DMSO vs ETV5_OCA	0.0226
Figure 2F	$T_{H}0$_DMSO vs $T_{H}0$_OCA	0.0119
Figure 2F	T_{FH}_DMSO vs T_{FH}_OCA	0.0002
Figure 2G	DMSO vs OCA	0.0010
Figure 2J	DMSO vs OCA	0.0022
Figure 2J	DMSO vs OPN	0.0369
Figure 2J	OCA vs OCA+OPN	0.0046
Figure 3D	WT_DMSO in Total IgG<br>vs WT_OCA in Total IgG	0.0001
Figure 3D	WT_DMSO in Total IgG<br>vs cKO_DMSO in Total IgG	0.0004
Figure 3D	WT_DMSO in Total IgG<br>vs cKO_OCA in Total IgG	0.0001
Figure 3D	WT_DMSO in IgG2c<br>vs WT_OCA in IgG2c	<0.0001
Figure 3D	WT_DMSO in IgG2c<br>vs cKO_DMSO in IgG2c	<0.0001
Figure 3D	WT_DMSO in IgG2c<br>vs cKO_OCA in IgG2c	<0.0001
Figure 3D	WT_DMSO in IgM<br>vs WT_OCA in IgM	0.0033

Figure 3D	WT_DMSO in IgM vs cKO_DMSO in IgM	0.0110
Figure 3E	WT_DMSO in Anti-dsDNA IgG vs WT_OCA in Anti-dsDNA IgG	0.0091
Figure 3E	WT_DMSO in Anti-dsDNA IgM vs WT_OCA in Anti-dsDNA IgM	0.0001
Figure 3E	WT_DMSO in Anti-dsDNA IgM vs cKO_DMSO in Anti-dsDNA IgM	0.0009
Figure 3E	WT_DMSO in Anti-dsDNA IgM vs cKO_OCA in Anti-dsDNA IgM	0.0047
Figure 3F	WT_DMSO in T_{FH} vs WT_OCA in T_{FH}	0.0006
Figure 3F	WT_DMSO in T_{FH} vs cKO_DMSO in T_{FH}	0.0059
Figure 3F	WT_DMSO in T_{FH} vs cKO_OCA in T_{FH}	0.0091
Figure 3F	WT_DMSO in T_{FR} vs WT_OCA in T_{FR}	0.0344
Figure 3G	WT_DMSO vs WT_OCA	0.0240
Figure 3G	WT_DMSO vs cKO_DMSO	0.0005
Figure 3H	WT_DMSO vs WT_OCA	0.0039
Figure 3H	WT_DMSO vs cKO_DMSO	0.0025
Figure 3H	WT_DMSO vs cKO_OCA	0.0497
Figure 4D	WT_DMSO in Total IgG vs *San*_DMSO in Total IgG	<0.0001
Figure 4D	*San*_DMSO in Total IgG vs *San*_OCA in Total IgG	0.0011
Figure 4D	WT_DMSO in Total IgG vs *San*_OCA in Total IgG	0.0007
Figure 4D	WT_DMSO in IgG2c vs *San*_DMSO in IgG2c	<0.0001
Figure 4D	*San*_DMSO in IgG2c	0.0013

	vs *San*_OCA in IgG2c	
Figure 4D	WT_DMSO in IgG2c vs *San*_OCA in IgG2c	0.0028
Figure 4D	WT_DMSO in IgM vs *San*_DMSO in IgM	<0.0001
Figure 4D	*San*_DMSO in IgM vs *San*_OCA in IgM	0.0007
Figure 4D	WT_DMSO in IgM vs *San*_OCA in IgM	0.0092
Figure 4D	WT_DMSO in IgG1 vs *San*_DMSO in IgG1	<0.0001
Figure 4D	*San*_DMSO in IgG1 vs *San*_OCA in IgG1	0.0007
Figure 4D	WT_DMSO in IgG1 vs *San*_OCA in IgG1	0.0091
Figure 4E	WT_DMSO in Anti-dsDNA IgG vs *San*_DMSO in Anti-dsDNA IgG	0.0005
Figure 4E	*San*_DMSO in Anti-dsDNA IgG vs *San*_OCA in Anti-dsDNA IgG	0.0197
Figure 4E	WT_DMSO in Anti-dsDNA IgG vs *San*_OCA in Anti-dsDNA IgG	0.0023
Figure 4E	WT_DMSO in Anti-dsDNA IgG2c vs *San*_DMSO in Anti-dsDNA IgG2c	0.0032
Figure 4E	*San*_DMSO in Anti-dsDNA IgG2c vs *San*_OCA in Anti-dsDNA IgG2c	0.0177
Figure 4E	WT_DMSO in Anti-dsDNA IgG2c vs *San*_OCA in Anti-dsDNA IgG2c	0.0049
Figure 4E	WT_DMSO in Anti-dsDNA IgM vs *San*_DMSO in Anti-dsDNA IgM	<0.0001
Figure 4E	*San*_DMSO in Anti-dsDNA IgM vs *San*_OCA in Anti-dsDNA IgM	0.0009

Figure 4F	WT_DMSO vs *San*_DMSO	0.0005
Figure 4F	*San*_DMSO vs *San*_OCA	0.0005
Figure 4G	WT_DMSO in T_{FH} vs *San*_DMSO in T_{FH}	<0.0001
Figure 4G	*San*_DMSO in T_{FH} vs *San*_OCA in T_{FH}	0.0001
Figure 4G	WT_DMSO in T_{FH} vs *San*_OCA in T_{FH}	<0.0001
Figure 4G	WT_DMSO in T_{FR} vs *San*_DMSO in T_{FR}	0.0033
Figure 4G	WT_DMSO in T_{FR} vs *San*_OCA in T_{FR}	0.0151
Figure 4H	WT_DMSO vs *San*_DMSO	<0.0001
Figure 4H	*San*_DMSO vs *San*_OCA	0.0234
Figure 4H	WT_DMSO vs *San*_OCA	0.0022
Figure 4I	WT_DMSO vs *San*_DMSO	<0.0001
Figure 4I	*San*_DMSO vs *San*_OCA	0.0011
Figure 4I	WT_DMSO vs *San*_OCA	0.0019
Figure EV1F	T_H0_DMSO vs T_H0_OCA	0.0483
Figure EV1F	T_H2_DMSO vs T_H2_OCA	0.0015
Figure EV1G	T_H17_DMSO vs T_H17_OCA	0.0157
Figure EV2A	DMSO vs OCA	0.0161
Figure EV2B	DMSO vs OCA	0.0494
Figure EV2C	DMSO vs OCA	0.0167
Figure EV3A	WT_DMSO in IgG2c vs WT_OCA in IgG2c	0.0124
Figure EV3A	cKO_DMSO in IgG2c vs cKO_OCA in IgG2c	0.0330
Figure EV3A	WT_DMSO in IgM vs WT_OCA in IgM	0.0067
Figure EV3A	cKO_DMSO in IgM vs cKO_OCA in IgM	0.0448
Figure EV4A	WT_DMSO vs cKO_DMSO	0.0234
Figure EV4A	WT_DMSO vs cKO_OCA	0.0083

Figure EV4B	WT_DMSO vs WT_OCA	0.0362
Figure EV4B	WT_DMSO vs cKO_DMSO	0.0242
Figure EV4B	WT_DMSO vs cKO_OCA	0.0489
Figure EV5A	WT_DMSO in $CD25^{+}Foxp3^{+}$ vs *San*_DMSO in $CD25^{+}Foxp3^{+}$	<0.0001
Figure EV5A	WT_DMSO in $CD25^{+}Foxp3^{+}$ vs *San*_OCA in $CD25^{+}Foxp3^{+}$	0.0423
Figure EV5A	WT_DMSO in $CD25^{-}Foxp3^{+}$ vs *San*_DMSO in $CD25^{-}Foxp3^{+}$	0.0290
Figure EV5B	WT_DMSO vs *San*_DMSO	<0.0001
Figure EV5B	WT_DMSO vs *San*_OCA	0.0002
Figure EV5C	WT_DMSO vs *San*_DMSO	<0.0001
Figure EV5C	WT_DMSO vs *San*_OCA	0.0002
Figure EV5D	WT_DMSO vs *San*_OCA	0.0424
