# Supplementary material for: Obeticholic acid alleviates lupus pathology by inhibiting T follicular helper cell differentiation
Source: EMBO Mol Med. 2026 Jul 3;18(8):3258–77. doi: 10.1038/s44321-026-00478-6 (PMC13469628; doi:10.1038/s44321-026-00478-6)
Supplement: Supplementary file 3 [file 44321_2026_478_MOESM2_ESM.pdf]

EMBO Molecular Medicine

Obeticholic acid alleviates lupus pathology by inhibiting T follicular helper cell differentiation

Yoontae Lee, Minjung Kang, Jiho Park, Jongeun Lee, and Sung Won Kim

Corresponding author: Yoontae Lee (yoontael@postech.ac.kr)

Review Timeline:

Submission Date:	11th Jan 26
Editorial Decision:	18th Feb 26
Revision Received:	18th May 26
Editorial Decision:	10th Jun 26
Revision Received:	14th Jun 26
Accepted:	19th Jun 26

Editor: Zeljko Durdevic

Transaction Report:

(Note: With the exception of the correction of typographical or spelling errors that could be a source of ambiguity, letters and reports are not edited. Depending on transfer agreements, referee reports obtained elsewhere may or may not be included in this compilation. Referee reports are anonymous unless the Referee chooses to sign their reports.)

18th Feb 2026

Dear Prof. Lee,

Thank you for the submission of your manuscript to EMBO Molecular Medicine. We have now received feedback from the three reviewers who agreed to evaluate your manuscript. All three referees recognize interest of the study but also raise serious and largely overlapping concerns that should be addressed in a major revision. If you would like to discuss further the points raised by the referees, I am available to do so via email or video. Let me know if you are interested in this option.

Further consideration of a revision that addresses reviewers' concerns in full will entail a second round of review. EMBO Molecular Medicine encourages a single round of revision only and therefore, acceptance or rejection of the manuscript will depend on the completeness of your responses included in the next, final version of the manuscript. For this reason, and to save you from any frustrations in the end, I would strongly advise against returning an incomplete revision.

We would welcome the submission of a revised version within three months for further consideration. Please let us know if you require longer to complete the revision.

I look forward to receiving your revised manuscript.

Yours sincerely,

Zeljko Durdevic

Zeljko Durdevic
Senior Editor
EMBO Molecular Medicine

*****

When preparing your revised manuscript, please refer to our guidelines: https://link.springer.com/journal/44321/submission-guidelines#cms-Revised-submissions. We perform an initial quality control of all revised manuscripts before re-review; failure to include requested items will delay the evaluation of your revision.

We require:

1) A .docx formatted version of the manuscript text (including legends for main figures, EV figures and tables). Please make sure that the changes are highlighted to be clearly visible.

2) Individual production quality figure files as .eps, .tif, .jpg (one file per figure). For guidance, download the 'Figure Guide PDF': https://media.springernature.com/original/springer-cms/rest/v1/content/27825798/data/v1.

3) A .docx formatted letter INCLUDING the reviewers' reports and your detailed point-by-point responses to their comments. As part of the EMBO Press transparent editorial process, the point-by-point response is part of the Review Process File (RPF), which will be published alongside your paper.

4) A complete author checklist, which you can download from our author guidelines. Please insert information in the checklist that is also reflected in the manuscript. The completed author checklist will also be part of the RPF.

5) Please note that all corresponding authors are required to supply an ORCID ID for their name upon submission of a revised manuscript.

6) It is mandatory to include a 'Data Availability' section after the Materials and Methods. Before submitting your revision, primary datasets produced in this study need to be deposited in an appropriate public database, and the accession numbers and database listed under 'Data Availability'. Please remember to provide a reviewer password if the datasets are not yet public.

In case you have no data that requires deposition in a public database, please state so in this section. Note that the Data Availability Section is restricted to new primary data that are part of this study.

7) For data quantification: please specify the name of the statistical test used to generate error bars and P values, the number (n) of independent experiments (specify technical or biological replicates) underlying each data point and the test used to calculate p-values in each figure legend. The figure legends should contain a basic description of n, P and the test applied. Graphs must include a description of the bars and the error bars (s.d., s.e.m.).

8) At EMBO Press we ask authors to provide source data for the main manuscript figures. You will receive a separate email with instructions for providing source data with your revised manuscript, including how to upload and organize the files.

9) Our journal encourages inclusion of *data citations in the reference list* to directly cite datasets that were re-used and obtained from public databases. Data citations in the article text are distinct from normal bibliographical citations and should directly link to the database records from which the data can be accessed. In the main text, data citations are formatted as follows: "Data ref: Smith et al, 2001" or "Data ref: NCBI Sequence Read Archive PRJNA342805, 2017". In the Reference list, data citations must be labeled with "[DATASET]". A data reference must provide the database name, accession number/identifiers and a resolvable link to the landing page from which the data can be accessed at the end of the reference.

10) We replaced Supplementary Information with Expanded View (EV) Figures and Tables that are collapsible/expandable online. A maximum of 5 EV Figures can be typeset. EV Figures should be cited as 'Figure EV1, Figure EV2" etc... in the text and their respective legends should be included in the main text after the legends of regular figures.

- For the figures that you do NOT wish to display as Expanded View figures, they should be bundled together with their legends in a single PDF file called *Appendix*, which should start with a short Table of Content. Appendix figures should be referred to in the main text as: "Appendix Figure S1, Appendix Figure S2" etc.

- Additional Tables/Datasets should be labeled and referred to as Table EV1, Dataset EV1, etc. Legends have to be provided in a separate tab in case of .xls files. Alternatively, the legend can be supplied as a separate text file (README) and zipped together with the Table/Dataset file.

11) The paper explained: EMBO Molecular Medicine articles are accompanied by a summary of the articles to emphasize the major findings in the paper and their medical implications for the non-specialist reader. Please provide a draft summary of your article highlighting

- the medical issue you are addressing,

- the results obtained and

- their clinical impact.

This may be edited to ensure that readers understand the significance and context of the research. Please refer to any of our published articles for an example.

12) Author contributions: You will be asked to provide CRediT (Contributor Role Taxonomy) terms in the submission system. These replace a narrative author contribution section in the manuscript.

13) A Conflict of Interest statement should be provided in the main text.

14) Every published paper includes a 'Synopsis' to further enhance discoverability. Synopses are displayed on the journal webpage and are freely accessible to all readers. They include a short stand first (maximum of 300 characters, including space) as well as 2-5 one-sentences bullet points that summarizes the paper. Please write the bullet points to summarize the key NEW findings. They should be designed to be complementary to the abstract - i.e. not repeat the same text. We encourage inclusion of key acronyms and quantitative information (maximum of 30 words / bullet point). Please use the passive voice. Please attach these in a separate file or send them by email, we will incorporate them accordingly.

Please also suggest a striking image or visual abstract to illustrate your article as a PNG file 550 px wide x 300-600 px high.

15) Include a Reagents and Tools Table as part of the Methods section, which can be downloaded from our author guidelines.

EMBO Molecular Medicine has a "scooping protection" policy, whereby similar findings that are published by others during review or revision are not a criterion for rejection. Should you decide to submit a revised version, I do ask that you get in touch after three months if you have not completed it, to update us on the status.

***** Reviewer's comments *****

Referee #1 (Remarks for Author):

This manuscript investigates the therapeutic potential of obeticholic acid (OCA), originally approved for primary biliary cholangitis, in the context of systemic lupus erythematosus (SLE). The authors propose that OCA alleviates lupus-like pathology by suppressing T follicular helper (Tfh) cell differentiation and subsequent germinal center (GC) B cell responses. Mechanistically, the study suggests that OCA downregulates the ETV5-Spp1 axis, which is critical for the Tfh program. These findings are supported by robust genetic models, including a T-cell-specific knockout line, CD4creEtv5fl/fl, and the Sanroque lupus mouse model.
The manuscript is well-written and the repositioning of OCA for SLE is a compelling prospect. However, the following major points need be addressed to strengthen the mechanistic and clinical conclusions.

1. Receptor Specificity of OCA in T cells
The identity of the specific receptor mediating OCA's effects in T cells remains unclear. As a bile acid analogue, OCA is a potent agonist of the Farnesoid X receptor (FXR). However, recent literature (e.g., Wang et al., Cell Mol Immunol, 2025) suggests that FXR signaling actually promotes Tfh programs in lupus. This presents a contradiction if OCA activates FXR, why is an inhibitory effect observed in this study? The authors should perform additional experiments to clarify this issue, or at least discuss this potentially controversial point in the manuscript.

2. Tissue-Specific Immunomodulation
Bile acids and their analogues exist at significantly higher concentrations within the enterohepatic circulation than in systemic lymphoid tissues. It is important to determine whether the observed effects are consistent across different anatomical sites. Thus it would be interesting to investigate whether Tfh populations in the spleen, Peyer's patches, and mesenteric lymph nodes are regulated similarly by OCA, or if the drug's efficacy is dependent on local tissue concentrations.

Referee #2 (Remarks for Author):

In this paper by Kang and coauthors, the authors study the impact of Obeticholac acid, which may impact ETV5 function, and subsequently inhibit Tfh differentiation and function. The paper demonstrate some extent of mechanistics, relies on several models and is well thought/presented. I have several comments to improve the solidity of the findings.

Major point:
The mechanistic claim is, as is, overclaimed as causality is not demonstrated.
1/ OCA inhibits ETV5 DNA binding is shown through CHIP-qPCR but it is not shown whether ETV5 protein level is affected by OCA. Please add at least a few WB experiments to confirm this. Showing nuclear localization or not of ETV5 under different condition would be a plus.
2/ The link between SPP1/OPN on the Tfh differentiation is shown be not causally demonstrated. If the authors would like to keep causal wording, they need to rescue spp1 and/or OPN expression in mice treated by OCA to rescue Tfh differentiation.
I understand these are complicate experiments but if the authors do not do that, they need to tone down all causality claim since OCA may have multiple impact on T cells outside only SPP1/OPN

Detailed comments:
1/ Figure 1:
- The in vitro Tfh differentiation plot is not very convincing. The authors should show Bcl6 expression on top of the PD1/CXCR1 expression and/or IL21 protein production (qPCR or cytokine expression using cytometry).
- Fig. 1B-D:
o it is unclear why the authors used the OVA-Alum models which induces a small Tfh expansion compared to other well described models : ie, Sheep Red Blood Cells. Here the differences are small but appear to exists.
o Since the authors used the OVA models, they can show the antibody production by measurement of the anti-OVA Ab in these conditions.
o It would be useful to show the absolute number of splenic cells on top of %
o Please show (in Fig EV2) other T cell subpopulations (Th1,2, 17, Treg) in the spleen in the 4 conditions to show the specificity of this intervention on Tfh/Tfr or if the sames differences are seen as in in vitro differentiation. In figure EV2 only 2 conditions appear.

o Fig 1E-G please provide also the absolute numbers.

2/ Figure 2:
- Please show colors for the different conditions (ie, Fig 2A) since its difficult to differentiate the conditions as is.
- Fig 2A-B : it would be more convincing if the authors showed other genes that are not affected by OCA (ie, pdcd1, PD1 protein).
- It is a bit destabilizing that the authors show first spp1 mRNA levels then OPN protein expression. Please shown opn mRNA expression too to be sure this change is affected on the transcription level.
- Fig 2E : usually human Th differentiation is 7 days. The authors evaluated cells at day 3 based on the legend ? Please clarify.

3/ Figure 3 and Figure 4
- Please also provide absolute numbers of subpopulation in the spleen.
- Fig 3C/4C: the pathology is very small. It would be useful to have a pathologist compare objectively (blinded) the conditions). What about proteinuria ?

Minor points:
- "These cells express BCL6, CXCR5, ICOS, and PD-1, and secrete IL-21, all of which facilitate their localization to B-cell follicles, enabling them to provide signals to GC B cells". Is not very clear : IL21 for example is an effector cytokines, it does not facilitate Tfh location to B cell follicles. This is driven by CXCR5. Please clarify.

Referee #3 (Remarks for Author):

In this manuscript, Kang and colleagues investigate the preclinical potential of repurposing obeticholic acid (OCA), a drug currently approved for liver disease, for the treatment of Tfh-driven autoimmunity. The authors propose that OCA inhibits Tfh cell differentiation through repression of ETV5 activity, thereby reducing Spp1-driven Tfh differentiation and ameliorating disease in two murine lupus models. The study first demonstrates that OCA reduces Tfh differentiation in human and murine in vitro systems, followed by in vivo validation using conditional ETV5-deficient mice, which exhibit marked reductions in Tfh-related phenotypes following immunisation, which the authors are able to recapitulate upon OCA treatment. Finally, OCA is evaluated in the pristane-induced and Sanroque preclinical mouse models of lupus, where treatment partially ameliorates serological, cellular and histological biomarkers of autoimmunity.

While the manuscript and data are interesting and important to the field, as the repurposing of an existing drug for the treatment of lupus would represent a significant step forward into discovering much-needed therapies, some concerns need to be addressed before the mechanistic claims can be fully supported.

1. My main concern is the overall conclusion that OCA treatment works through ETV5, which is only partially supported by the data.
The use of OCA-treated ETV5 cKO animals demonstrates that pharmacological inhibition phenocopies genetic ablation. This convincingly shows that OCA is sufficient to reduce Tfh numbers to a similar extent as ETV5 deficiency. However, phenocopy alone does not establish i) causation or ii) specificity.
i) To the former point, and to demonstrate causation, the authors should have a system where ETV5 is overactive (and they have this system in Fig 1E (Cic cKO), but do not use it in their preclinical mouse models of autoimmunity. The authors should either, evaluate the markers of autoimmunity in pristane and/or sanroque models, to ascertain that OCA treatment does indeed restore the autoimmune phenotypes conferred by ETV5 overactivity in this model, or to soften the claim that OCA-driven abrogation of Tfh cells and autoimmunity is through ETV5.
ii) Regarding specificity, while the CHIP data on Fig 2c shows that OCA disrupts ETV5 binding to Spp1 and downstream expression, it remains unclear whether OCA broadly suppresses ETV5 genomic occupancy or selectively affects Spp1. The extent of genome-wide disruption is not defined. Therefore, the specificity of OCA-mediated repression of Tfh differentiation through Spp1 remains uncertain. Although the authors acknowledge this point to some extent in the discussion, they should articulate it more clearly throughout the manuscript.
2. Fig 1A: The flow cytometric profiles of in vitro-generated Tfh cells are not entirely convincing, and do not resemble those reported in the Gao et al 2023 Elife paper. The CXCR5+PD-1+ population appears uniformly distributed, and the gating strategy seems somewhat arbitrary. Given the uniform distribution of both PD-1 and CXCR5, it might be more appropriate to report the MFI expression for each marker separately and argue that OCA treatment reduced the expression of these markers.
3. The authors argue that OCA inactivates ETV5. Their experiments using ETV5 flox mice are convincing, but those using Cic

flox mice (Fig 1E), which exhibit de-repression of ETV5, are less so, as OCA treatment in these mice only partially rescued the aberrant expansion of Tfh cells by ~30%. This partial rescue raises two possibilities:
(i) OCA dosage may be insufficient to fully inhibit ETV5 activity in the setting of overexpression; or
(ii) OCA acts through both ETV5-dependent and ETV5-independent pathways.
The authors should explicitly acknowledge these alternative interpretations rather than implying complete mechanistic specificity.
4. The authors should acknowledge either the technical limitation of their study (i.e. the amount of OCA given cannot completely abrogate ETV5 activity if they have evidence for it) or that OCA also operates to repress Tfh differentiation at least partially through ETV5 and through other pathways too.
5. In Figure 4C it is strange that the authors report glomerulonephritis at 7 weeks of age. Sanroque mice typically develop renal pathology by ~ 6 months of age (PMID: 23159227), and the histological evidence presented here is not entirely convincing or robust enough to demonstrate immune infiltration. The authors should either: i) conduct the kidney clinical pathology scoring system found in the previous reference and present robust quantitative evidence; or ii) evaluate kidney pathology at 6 months of age. However, given the weight of the other data (anti nuclear antibodies and total circulating antibodies), which is solid and convincing, and if the proposed experiment is not feasible, I would suggest the authors remove the kidney pathology data.

Minor
1. References should be placed before the period.
2. In Figure 1E, given the relatively large sample size ($n \approx 8$), reporting medians rather than means may be more appropriate. The presence of two outliers in the "cKO OCA-" group appears to inflate the mean substantially.
3. P7, in the line "Because OCA suppresses the ETV5 activity by inhibiting its DNA binding,(Lee et al., 2025)" binding should read "DNA-binding domain"?

Point-by-point responses: our responses are written in red.

We sincerely thank the Editor and the Reviewers for their careful evaluation of our manuscript and for their constructive and insightful comments. We have revised the manuscript extensively in response to these suggestions. In particular, we performed additional experiments to strengthen the mechanistic basis of the study, including analysis of ETV5 protein expression, OPN rescue experiments, and assessment of tissue-specific T_{FH} cell responses in mesenteric lymph nodes and Peyer's patches. We also revised the manuscript throughout to moderate overinterpretation, clarify the limitations of the study, and improve the presentation of the data. Our detailed responses are provided below.

Reviewer #1:

1. Receptor Specificity of OCA in T cells

: The identity of the specific receptor mediating OCA's effects in T cells remains unclear. As a bile acid analogue, OCA is a potent agonist of the Farnesoid X receptor (FXR). However, recent literature (e.g., Wang et al., Cell Mol Immunol, 2025) suggests that FXR signaling actually promotes Tfh programs in lupus. This presents a contradiction if OCA activates FXR, why is an inhibitory effect observed in this study? The authors should perform additional experiments to clarify this issue, or at least discuss this potentially controversial point in the manuscript.

We thank the Reviewer for this important comment. We agree that the receptor specificity underlying the effect of OCA in T cells remains to be fully resolved. In response, we have substantially expanded the Discussion to address the potential contribution of FXR signaling and the apparent discrepancy with a recent report indicating that FXR promotes pathogenic T_{FH} responses in lupus. In the revised manuscript, we now explicitly state that although our data identify ETV5 as a key mediator of the inhibitory effect of OCA on T_{FH} cell differentiation, we cannot exclude the possibility that FXR also contributes to the overall response. We further emphasize that additional studies will be required to more precisely distinguish FXR-dependent from ETV5-specific pathways. These revisions more clearly acknowledge this mechanistic issue and avoid overstating the specificity of OCA toward ETV5.

2. Tissue-Specific Immunomodulation

: Bile acids and their analogues exist at significantly higher concentrations within the enterohepatic circulation than in systemic lymphoid tissues. It is important to determine whether the observed effects are consistent across different anatomical sites. Thus it would be interesting to investigate whether Tfh populations in the spleen, Peyer's patches, and mesenteric lymph nodes are regulated similarly by OCA, or if the drug's efficacy is dependent on local tissue concentrations.

We appreciate this helpful suggestion. To determine whether the effect of OCA is restricted to the spleen or extends to other anatomical sites, we analyzed T_{FH} cells in the mesenteric lymph nodes and Peyer's patches following OVA immunization. OCA significantly reduced T_{FH} cell frequencies in both tissues, as shown in revised Figure EV2A and B. In the same model, we also measured serum anti-OVA IgG and observed a significant reduction in the antigen-specific humoral response (revised Figure EV2C). Together, these data indicate that the inhibitory effect of OCA on T_{FH} cell responses is not confined to a specific lymphoid organ.

Reviewer #2:

Major point:

1/ OCA inhibits ETV5 DNA binding is shown through CHIP-qPCR but it is not shown whether ETV5 protein level is affected by OCA. Please add at least a few WB experiments to confirm this. Showing nuclear localization or not of ETV5 under different condition would be a plus.

We thank the Reviewer for this valuable suggestion. To address this point, we performed western blot analysis to assess ETV5 protein expression under T_{FH}-polarizing conditions in the presence of DMSO or OCA. As shown in revised Figure 2E, OCA did not alter ETV5 protein abundance. These data support the interpretation that OCA suppresses T_{FH} cell differentiation without detectably reducing ETV5 expression, consistent with an effect on ETV5 function rather than its abundance.

2/ The link between SPP1/OPN on the Tfh differentiation is shown be not causally demonstrated. If the authors would like to keep causal wording, they need to rescue spp1 and/or OPN expression in mice treated by OCA to rescue Tfh differentiation.

I understand these are complicate experiments but if the authors do not do that, they need to tone down all causality claim since OCA may have multiple impact on T cells outside only SPP1/OPN

We appreciate the Reviewer's insightful point and agree that a functional rescue experiment is critical to strengthen causality. In response, we performed an *in vivo* OPN rescue assay. In this experiment, recombinant OPN was administered together with OCA during OVA immunization, followed by analysis of splenic T_{FH} cell frequencies. As shown in revised Figure 2I and J, recombinant OPN restored T_{FH} cell differentiation suppressed by OCA treatment. These findings support a role for the ETV5–SPP1 axis as a key mediator of the inhibitory effect of OCA on T_{FH} cell differentiation *in vivo*. At the same time, we have revised the manuscript throughout to avoid overstatement and to acknowledge that additional ETV5-regulated targets may also contribute.

Detailed comments:

1/ Figure 1:

- The in vitro Tfh differentiation plot is not very convincing. The authors should show Bcl6 expression on top of the PD1/CXCR5 expression and/or IL21 protein production (qPCR or cytokine expression using cytometry).

We thank the Reviewer for this helpful suggestion. To strengthen this analysis, we expanded the flow cytometric assessment of the T_{FH} program. The revised Figure 1 now includes the frequency of $BCL6^+$ cells alongside the original $PD1^+CXCR5^+$ T_{FH}-like gate in Figure 1A, together with quantification of PD-1 mean fluorescence intensity (MFI) in Figure 1B, CXCR5 MFI in Figure 1C, and BCL6 MFI in Figure 1D. These additions provide a more quantitative and comprehensive evaluation of the effect of OCA on the T_{FH} program and directly address the concern that the original profile alone was not sufficiently convincing. Although IL-21 protein analysis was not included in this revision, the added BCL6-based analyses materially strengthen this part of the dataset.

- Fig. 1B-D:

o it is unclear why the authors used the OVA-Alum models which induces a small Tfh expansion compared to other well described models: ie, Sheep Red Blood Cells. Here the differences are small but appear to exists.

We appreciate the Reviewer's comment. We agree that SRBC is a robust model for eliciting T_{FH} cell responses. In the present study, however, we employed the OVA-alum model because it is a well-established T cell-dependent immunization system that enables direct evaluation of antigen-specific T_{FH} responses and antibody production. This model was particularly suited to our study. as it allowed us to assess antigen-specific T_{FH} responses under OCA treatment. To strengthen this aspect of the manuscript, we additionally measured serum anti-OVA IgG levels and found that OCA significantly reduced the antigen-specific humoral response in this model, as shown in revised Figure EV2C. We have clarified this rationale in the revised manuscript.

o Since the authors used the OVA models, they can show the antibody production by measurement of the anti-OVA Ab in these conditions.

We have now included an analysis of serum anti-OVA IgG in OVA-immunized mice treated with DMSO or OCA. OCA significantly reduced anti-OVA IgG levels, consistent with its inhibitory effect on T_{FH} cell responses, as shown in revised Figure EV2C.

o It would be useful to show the absolute number of splenic cells on top of %

We appreciate this comment. Unfortunately, these experiments were designed primarily for frequency-based flow cytometric analysis, and absolute total splenocyte counts were not recorded in a manner that allows accurate retrospective calculation of subset numbers. Therefore, we were not able to include absolute counts in the revision. Nevertheless, our main conclusions are supported by multiple independent lines of evidence, including T_{FH} and GC B cell frequencies and antigen-specific antibody responses.

o Please show (in Fig EV2) other T cell subpopulations (Th1,2, 17, Treg) in the spleen in the 4 conditions to show the specificity of this intervention on Tfh/Tfr or if the sames differences are seen as in in vitro differentiation. In figure EV2 only 2 conditions appear.

We thank the Reviewer for this helpful suggestion. In the revised manuscript, we have expanded Figure EV2 to include additional analyses of splenic $CD4^+$ T-cell subsets in OVA-immunized *Etv5*$^{f/f}$ and *Etv5*$^{f/f}$*;Cd4-Cre* mice treated with DMSO or OCA. Specifically, we now present data on effector $CD4^+$ T cells, T_{REG} cells, T_H1 cells, T_H2 cells, and T_H17 cells across the indicated conditions, as shown in revised Figure EV2D–K. These additional data further support the relative selectivity of OCA for the T_{FH} cell program *in vivo*.

o Fig 1E-G please provide also the absolute numbers.

The same limitation applies here. These experiments were not designed to preserve the information required for accurate retrospective calculation of absolute splenic subset counts, so we retained the frequency-based analyses that were acquired consistently across groups. We have kept the interpretation accordingly restrained. The overall conclusion remains supported by multiple complementary datasets, including the frequencies of T_{FH}, T_{FR}, and GC B cells.

2/ Figure 2:

- Please show colors for the different conditions (ie, Fig 2A) since its difficult to differentiate the conditions as is.

We thank the Reviewer for this practical suggestion. We revised the color scheme in the figure to improve visual distinction between the indicated conditions.

- Fig 2A-B : it would be more convincing if the authors showed other genes that are not affected by OCA (ie, pdcd1, PD1 protein).

We thank the Reviewer for this helpful suggestion. We addressed this point by assessing expression of *Cic*, a reported regulator of T_{FH} cell differentiation (Park *et al*, 2017), under T_{FH}-polarizing conditions with or without OCA treatment. *Cic* mRNA expression was not significantly changed by OCA (Figure R1). These data provide an additional negative control and support the interpretation that OCA does not broadly or nonspecifically suppress T_{FH}-related gene expression.

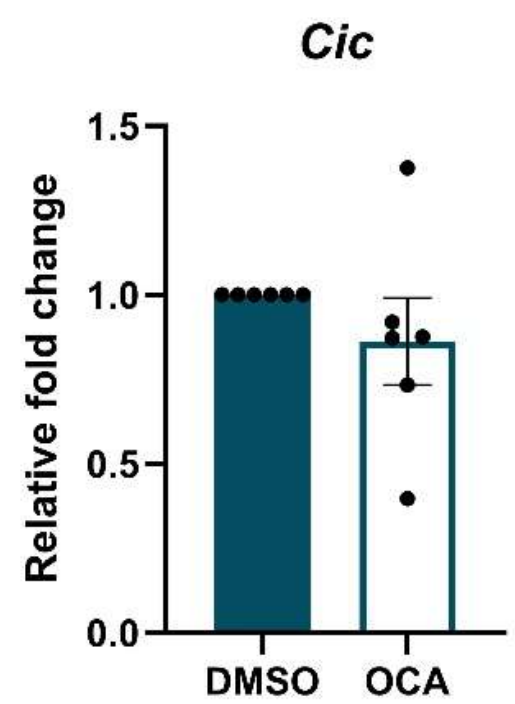

Figure R1. RT-qPCR analysis of *Cic* expression in mouse naïve CD4^{+} T cells cultured under T_{FH}-polarizing conditions in the presence of DMSO or OCA for 72 h (n = 6 per group). Data are representative of two independent experiments. Bar graphs present the data as mean ± SEM values. Statistical significance was determined using two-tailed unpaired Student's t-tests for the indicated pairwise comparisons. Each dot represents a biological replicate.

- It is a bit destabilizing that the authors show first spp1 mRNA levels then OPN protein expression. Please shown opn mRNA expression too to be sure this change is affected on the transcription level.

We thank the Reviewer for this comment. In mice, *Spp1* is the gene encoding osteopontin (OPN). Accordingly, the RT-qPCR data in Figure 2A represent transcriptional analysis of the OPN-encoding gene, while the ELISA in Figure 2B provides the corresponding protein-level measurement.

- Fig 2E : usually human Th differentiation is 7 days. The authors evaluated cells at day 3 based on the legend ? Please clarify.

We thank the Reviewer for this comment. The 3-day culture period used in our human T_{FH}-differentiation assay was based on previously published protocols (Kim *et al*, 2018; Locci *et al*, 2016) and our prior work (Park *et al*, 2024), in which early human T_{FH}-polarizing responses were assessed over 3–5 days. We therefore used a 3-day culture period in the present study.

3/ Figure 3 and Figure 4

- Please also provide absolute numbers of subpopulation in the spleen.

The same limitation applies to Figures 3 and 4. Unfortunately, these experiments were designed primarily for frequency-based flow cytometric analysis, and absolute total splenocyte counts were not recorded in a manner that allows accurate retrospective calculation of subset numbers. We have therefore limited the interpretation to what the dataset directly supports. The major conclusions remain supported by multiple independent lines of evidence, including T_{FH}, T_{FR}, and GC B cell frequencies.

- Fig 3C/4C: the pathology is very small. It would be useful to have a pathologist compare objectively (blinded) the conditions). What about proteinuria ?

We thank the Reviewer for this important comment. We agree that additional pathological assessment, such as blinded pathology scoring or proteinuria analysis, would further strengthen this part of the study. Although we were unable to perform these analyses within the scope of the current revision, we sought to improve transparency by adding Appendix Figures S1 and S2, which provide three additional representative H&E images per group for each tissue analyzed. We also revised the main text to moderate the pathological interpretation and now describe these findings more conservatively as reduced IgG deposition and amelioration of histopathological abnormalities. Importantly, the main conclusions of the study are supported not only by histological observations but also by consistent serological and immunological findings, including T_{FH} and GC B cell frequencies, serum immunoglobulin levels, anti-dsDNA antibodies, and serum OPN levels.

Minor points:

- "These cells express BCL6, CXCR5, ICOS, and PD-1, and secrete IL-21, all of which facilitate their localization to B-cell follicles, enabling them to provide signals to GC B cells". Is not very clear : IL21 for example is an effector cytokines, it does not facilitate Tfh location to B cell follicles. This is driven by CXCR5. Please clarify.

We thank the Reviewer for pointing this out. We revised the sentence to clarify that CXCR5 specifically facilitates localization of T_{FH} cells to B cell follicles, whereas IL-21 functions as an effector cytokine that supports GC B cell responses. The revised text is now more precise.

Reviewer #3

1. My main concern is the overall conclusion that OCA treatment works through ETV5, which is only partially supported by the data.

The use of OCA-treated ETV5 cKO animals demonstrates that pharmacological inhibition phenocopies genetic ablation. This convincingly shows that OCA is sufficient to reduce Tfh numbers to a similar extent as ETV5 deficiency. However, phenocopy alone does not establish i) causation or ii) specificity.

i) To the former point, and to demonstrate causation, the authors should have a system where ETV5 is overactive (and they have this system in Fig 1E (Cic cKO), but do not use it in their preclinical mouse models of autoimmunity. The authors should either, evaluate the markers of autoimmunity in pristane and/or sanroque models, to ascertain that OCA treatment does indeed restore the autoimmune phenotypes conferred by ETV5 overactivity in this model, or to soften the claim that OCA-driven abrogation of Tfh cells and autoimmunity is through ETV5.

We appreciate this important point. To address this concern, we expanded our analysis in T cell-specific *Cic*-deficient mice, in which ETV5 is derepressed. In this model, OCA significantly reduced T_{FH}, T_{FR}, and GC B cell frequencies, and also reduced serum IgG2c and IgM levels, with total IgG showing a decreasing trend, as shown in revised Figure 1H–J; EV3A. Because these mice were analyzed at a relatively early disease stage in the present study (Park *et al.*, 2024; Park *et al.*, 2017), we focused primarily on the ability of OCA to suppress T_{FH}-associated responses under conditions of ETV5 derepression.

ii) Regarding specificity, while the CHIP data on Fig 2c shows that OCA disrupts ETV5 binding to Spp1 and downstream expression, it remains unclear whether OCA broadly suppresses ETV5 genomic occupancy or selectively affects Spp1. The extent of genome-wide disruption is not defined. Therefore, the specificity of OCA-mediated repression of Tfh differentiation through Spp1 remains uncertain. Although the authors acknowledge this point to some extent in the discussion, they should articulate it more clearly throughout the manuscript.

We thank the Reviewer for this important point. We agree that our ChIP-qPCR data demonstrate reduced ETV5 binding at the *Spp1* promoter, but do not define the genome-wide extent of OCA-mediated effects on ETV5 chromatin binding. To address this concern, we have revised the manuscript at two levels. First, we added an *in vivo* OPN rescue experiment,

showing that recombinant OPN restored T_{FH} cell differentiation suppressed by OCA (revised Figure 2I and J). This provides functional evidence that the ETV5–SPP1 axis is an important mediator of the response. Second, we expanded the Discussion to explicitly state that the present study does not determine whether OCA broadly alters ETV5 binding across the genome or preferentially affects a subset of ETV5 targets, and that additional ETV5-regulated genes may also contribute. We believe these changes more accurately reflect the scope and limitations of the current data.

2. Fig 1A: The flow cytometric profiles of in vitro-generated Tfh cells are not entirely convincing, and do not resemble those reported in the Gao et al 2023 Elife paper. The CXCR5+PD-1+ population appears uniformly distributed, and the gating strategy seems somewhat arbitrary. Given the uniform distribution of both PD-1 and CXCR5, it might be more appropriate to report the MFI expression for each marker separately and argue that OCA treatment reduced the expression of these markers.

We thank the Reviewer for this constructive suggestion. In response, we strengthened this analysis by expanding the *in vitro* OT-II T_{FH}-like differentiation dataset. The revised Figure 1 now includes the frequency of $BCL6^{+}$ cells together with quantification of PD-1 MFI, CXCR5 MFI, and BCL6 MFI (revised Figure 1A–D). These additional data provide a more quantitative assessment of the effect of OCA on the T_{FH}-associated phenotype and directly address the concern regarding reliance on the original $CXCR5^{+}PD\text{-}1^{+}$ gate alone.

3. The authors argue that OCA inactivates ETV5. Their experiments using ETV5 flox mice are convincing, but those using Cic flox mice (Fig 1E), which exhibit de-repression of ETV5, are less so, as OCA treatment in these mice only partially rescued the aberrant expansion of Tfh cells by ~30%. This partial rescue raises two possibilities:

(i) OCA dosage may be insufficient to fully inhibit ETV5 activity in the setting of overexpression; or

(ii) OCA acts through both ETV5-dependent and ETV5-independent pathways.

The authors should explicitly acknowledge these alternative interpretations rather than implying complete mechanistic specificity.

We appreciate this important point. We agree that the effect of OCA in *Cic*-deficient mice is

partial rather than complete. To reflect this more accurately, we revised the manuscript to avoid implying complete mechanistic specificity. The revised text now states that OCA suppresses T_{FH}-associated responses under conditions of ETV5 derepression, albeit incompletely. We chose this wording to better reflect the partial nature of the response, which is consistent with our previous findings that T_{FH} cell responses were not fully abolished even in *Etv5*-deficient or *Cic/Etv5* double-deficient mice (Park *et al.*, 2024).

4. The authors should acknowledge either the technical limitation of their study (i.e. the amount of OCA given cannot completely abrogate ETV5 activity if they have evidence for it) or that OCA also operates to repress Tfh differentiation at least partially through ETV5 and through other pathways too.

We thank the Reviewer for this suggestion and agree. We have revised the manuscript to acknowledge this limitation more explicitly. Specifically, we now avoid language implying complete specificity and instead state that OCA acts predominantly through ETV5-mediated mechanisms. We also note that the incomplete rescue observed under conditions of ETV5 derepression is consistent with our previous findings that T_{FH} cell responses were reduced, but not fully abolished, even in *Etv5*-deficient or *Cic/Etv5* double-deficient mice (Park *et al.*, 2024).

5. In Figure 4C it is strange that the authors report glomerulonephritis at 7 weeks of age. Sanroque mice typically develop renal pathology by ~ 6 months of age (PMID: 23159227), and the histological evidence presented here is not entirely convincing or robust enough to demonstrate immune infiltration. The authors should either: i) conduct the kidney clinical pathology scoring system found in the previous reference and present robust quantitative evidence; or ii) evaluate kidney pathology at 6 months of age. However, given the weight of the other data (anti nuclear antibodies and total circulating antibodies), which is solid and convincing, and if the proposed experiment is not feasible, I would suggest the authors remove the kidney pathology data.

We thank the Reviewer for this important comment. We agree that renal pathology in *Sanroque* mice should be interpreted cautiously, particularly at relatively early time points. However, our analysis was performed at 10 weeks of age, which is later than the 8-week time point examined in previous work (Park *et al.*, 2024). Although we were unable to perform

formal kidney pathology scoring or extend the analysis to older mice within the scope of the current revision, we addressed this concern in two ways. First, we revised the manuscript to moderate the interpretation of the pathology data in the *Sanroque* model and removed stronger pathological wording. The revised text now refers more conservatively to reduced IgG deposition and amelioration of histopathological abnormalities. Second, we added Appendix Figure S2 showing three additional representative H&E images per group to improve transparency and allow broader visual assessment of the tissue findings. Importantly, the principal conclusions of the manuscript rely primarily on the consistent serological and immunological phenotypes, including T_{FH} and GC B cell responses, circulating immunoglobulins, anti-dsDNA antibodies, and serum OPN levels.

Minor

1. References should be placed before the period.

We thank the Reviewer for noting this point. We revised the manuscript accordingly and ensured that references are placed before the period throughout the text.

2. In Figure 1E, given the relatively large sample size (n ≈ 8), reporting medians rather than means may be more appropriate. The presence of two outliers in the "cKO OCA-" group appears to inflate the mean substantially.

We appreciate the Reviewer's careful observation. We re-analyzed this panel using a median-based approach and found that the overall interpretation remained unchanged (Figure R2). This result indicates that the conclusion is not dependent on the use of mean values or driven by outlier data points. For consistency with the presentation used throughout the manuscript, we retained the original format in the figure.

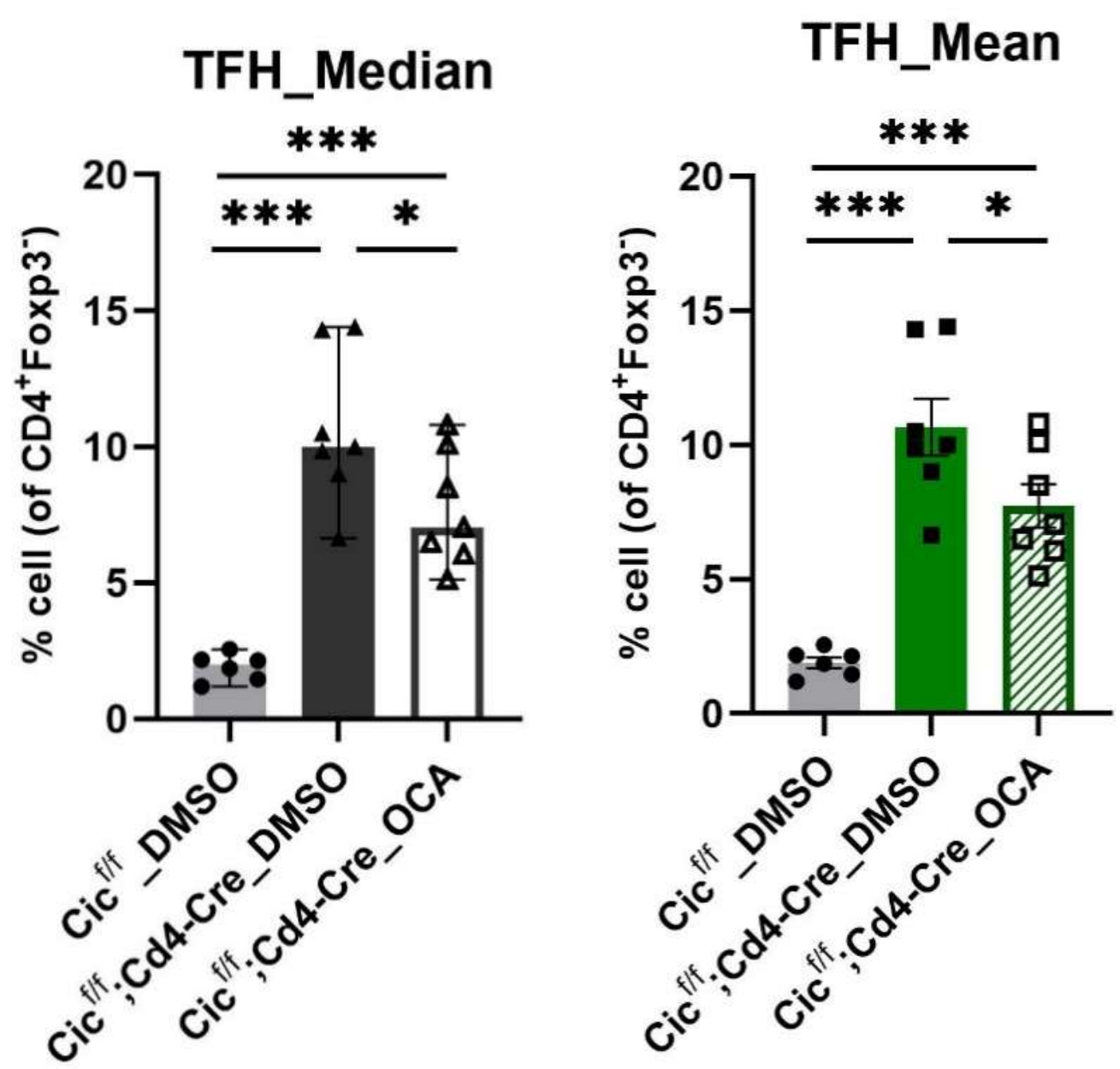

Figure R2. Flow cytometry analysis of T_{FH} cells in twenty-four-week-old *Cic^f/f^* (WT) and *Cic^f/f^;Cd4-Cre* (cKO) mice intravenously administered DMSO (vehicle) or OCA every three days for 27 days (WT, n = 6 and cKO, n = 7). Data are representative of three independent experiments. Left bar graphs display the median values for visualization, whereas right bar graphs display the mean ± SEM. Statistical significance was determined using two-tailed unpaired Student's t-tests for the indicated pairwise comparisons. Each dot represents a biological replicate.

3. P7, in the line "Because OCA suppresses the ETV5 activity by inhibiting its DNA binding,(Lee et al., 2025)" binding should read "DNA-binding domain"?

We thank the Reviewer for this comment. Our intention was to refer to inhibition of ETV5 DNA-binding activity, rather than the DNA-binding domain itself, and we have clarified this interpretation in the revised manuscript.

Reference

Kim CJ, Lee CG, Jung JY, Ghosh A, Hasan SN, Hwang SM, Kang H, Lee C, Kim GC, Rudra D *et al* (2018) The Transcription Factor Ets1 Suppresses T Follicular Helper Type 2 Cell Differentiation to Halt the Onset of Systemic Lupus Erythematosus. *Immunity* 49: 1034–1048 e1038

Locci M, Wu JE, Arumemi F, Mikulski Z, Dahlberg C, Miller AT, Crotty S (2016) Activin A programs the differentiation of human TFH cells. *Nat Immunol* 17: 976–984

Park J, Lee J, Hur Y, Kim CJ, Kim HB, Um D, Kim DS, Lee JY, Park S, Park Y *et al* (2024) ETV5 promotes lupus pathogenesis and follicular helper T cell differentiation by inducing osteopontin expression. *Proc Natl Acad Sci U S A* 121: e2322009121

Park S, Lee S, Lee CG, Park GY, Hong H, Lee JS, Kim YM, Lee SB, Hwang D, Choi YS *et al* (2017) Capicua deficiency induces autoimmunity and promotes follicular helper T cell differentiation via derepression of ETV5. *Nat Commun* 8: 16037

10th Jun 2026

Dear Prof. Lee,

Thank you for submitting your revised manuscript to EMBO Molecular Medicine. Your manuscript will be ready for acceptance pending the following final amendments:

1) Figures: We note that in Appendix Figure S2A San/San DMSO first and second image from the top are the same with slightly changed field of view. Please remove one of the images or replace it with another representative image from a distinct field of view.
2) In the main manuscript file, please do the following:
- Please address all comments suggested by our data editors listed below:
o Data availability statement:
1. Please note that the specific URL for S-BIAD3311 dataset is not provided in the data availability statement.
2. Please note that the database name for the S-BIAD3311dataset is not provided in the data availability statement.
o Figure legends:
1. Please note that the exact p values are not provided in the legends of figures 1A, D, G, J; 2B, 3D, 4D, E, G, H, I; EV5 A-C.
- Please correct the order of the sections in the manuscript text to: Abstract / The Paper Explained / Introduction / Results / Discussion / Methods / Data Availability / Acknowledgements / Disclosure and Competing Interests Statement / References / Figure Legends (if applicable) / Expanded View Figure Legends
- Rename "Methods and Protocols" to "Methods". Please remove the heading "Method details".
- In Methods, provide the antibody dilutions that were used for each antibody.
- Indicate in legends number and nature of replicates and exact p= values, not a range, along with the statistical test used. To keep the figures "clear" some authors found providing an Appendix table Sx with all exact p-values preferable. You are welcome to do this if you want to.
- Author contributions: Please remove it from the manuscript and specify author contributions in our submission system. CRediT has replaced the traditional author contributions section because it offers a systematic machine-readable author contributions format that allows for more effective research assessment. You are encouraged to use the free text boxes beneath each contributing author's name to add specific details on the author's contribution. More information is available in our guide to authors:
https://www.embopress.org/page/journal/17574684/authorguide#authorshipguidelines
- Please use the following format to report the accession number of your data:

The datasets produced in this study are available in the following databases:
[data type]: [full name of the resource] [accession number/identifier] ([doi or URL or identifiers.org/DATABASE:ACCESSION])

Please check "Author Guidelines" for more information.
https://www.embopress.org/page/journal/17574684/authorguide#availabilityofpublishedmaterial
3) The Paper Explained: Please add it to the main manuscript text.
4) Synopsis:
- Synopsis image: Please resize the image to exactly 550 px-wide x 300 pixels high.
- Please check your synopsis text and image before submission with your revised manuscript. Please be aware that in the proof stage minor corrections only are allowed (e.g., typos).
5) As part of the EMBO Publications transparent editorial process (see our Editorial at http://embomolmed.embopress.org/content/2/9/329), EMBO Molecular Medicine will publish online a Review Process File (RPF) to accompany accepted manuscripts. This file will be published in conjunction with your paper and will include the anonymous referee reports, your point-by-point response and all pertinent correspondence relating to the manuscript. Let us know if you want to remove or not any figures from it prior to publication. Please note that the Authors checklist will be published at the end of the RPF.
6) Please provide a point-by-point letter INCLUDING my comments as well as the reviewer's reports and your detailed responses (as Word file).

I look forward to reading a new revised version of your manuscript as soon as possible.

Yours sincerely,

Zeljko Durdevic

Zeljko Durdevic
Senior Editor

*** Instructions to submit your revised manuscript ***

*** PLEASE NOTE *** As part of the EMBO Publications transparent editorial process initiative (see our Editorial at https://www.embopress.org/doi/pdf/10.1002/emmm.201000094), EMBO Molecular Medicine will publish online a Review Process File to accompany accepted manuscripts.

In the event of acceptance, this file will be published in conjunction with your paper and will include the anonymous referee reports, your point-by-point response and all pertinent correspondence relating to the manuscript. If you do NOT want this file to be published, please inform the editorial office at contact@embomolmed.org.

*****

When preparing your revised manuscript, please refer to our guidelines: https://link.springer.com/journal/44321/submission-guidelines#cms-Revised-submissions. We perform an initial quality control of all revised manuscripts before re-review; failure to include requested items will delay the evaluation of your revision.

We require:

1) A .docx formatted version of the manuscript text (including legends for main figures, EV figures and tables). Please make sure that the changes are highlighted to be clearly visible.

2) Individual production quality figure files as .eps, .tif, .jpg (one file per figure). For guidance, download the 'Figure Guide PDF': https://media.springernature.com/original/springer-cms/rest/v1/content/27825798/data/v1.

3) A .docx formatted letter INCLUDING the reviewers' reports and your detailed point-by-point responses to their comments. As part of the EMBO Press transparent editorial process, the point-by-point response is part of the Review Process File (RPF), which will be published alongside your paper.

4) A complete author checklist, which you can download from our author guidelines. Please insert information in the checklist that is also reflected in the manuscript. The completed author checklist will also be part of the RPF.

5) Please note that all corresponding authors are required to supply an ORCID ID for their name upon submission of a revised manuscript.

6) It is mandatory to include a 'Data Availability' section after the Materials and Methods. Before submitting your revision, primary datasets produced in this study need to be deposited in an appropriate public database, and the accession numbers and database listed under 'Data Availability'. Please remember to provide a reviewer password if the datasets are not yet public.

In case you have no data that requires deposition in a public database, please state so in this section. Note that the Data Availability Section is restricted to new primary data that are part of this study.

7) For data quantification: please specify the name of the statistical test used to generate error bars and P values, the number (n) of independent experiments (specify technical or biological replicates) underlying each data point and the test used to calculate p-values in each figure legend. The figure legends should contain a basic description of n, P and the test applied. Graphs must include a description of the bars and the error bars (s.d., s.e.m.).

8) At EMBO Press we ask authors to provide source data for the main manuscript figures. You will receive a separate email with instructions for providing source data with your revised manuscript, including how to upload and organize the files.

9) Our journal encourages inclusion of *data citations in the reference list* to directly cite datasets that were re-used and obtained from public databases. Data citations in the article text are distinct from normal bibliographical citations and should directly link to the database records from which the data can be accessed. In the main text, data citations are formatted as follows: "Data ref: Smith et al, 2001" or "Data ref: NCBI Sequence Read Archive PRJNA342805, 2017". In the Reference list, data citations must be labeled with "[DATASET]". A data reference must provide the database name, accession

number/identifiers and a resolvable link to the landing page from which the data can be accessed at the end of the reference.

10) We replaced Supplementary Information with Expanded View (EV) Figures and Tables that are collapsible/expandable online. A maximum of 5 EV Figures can be typeset. EV Figures should be cited as 'Figure EV1, Figure EV2" etc... in the text and their respective legends should be included in the main text after the legends of regular figures.

- For the figures that you do NOT wish to display as Expanded View figures, they should be bundled together with their legends in a single PDF file called *Appendix*, which should start with a short Table of Content. Appendix figures should be referred to in the main text as: "Appendix Figure S1, Appendix Figure S2" etc.

- Additional Tables/Datasets should be labeled and referred to as Table EV1, Dataset EV1, etc. Legends have to be provided in a separate tab in case of .xls files. Alternatively, the legend can be supplied as a separate text file (README) and zipped together with the Table/Dataset file.

11) The paper explained: EMBO Molecular Medicine articles are accompanied by a summary of the articles to emphasize the major findings in the paper and their medical implications for the non-specialist reader. Please provide a draft summary of your article highlighting

- the medical issue you are addressing,

- the results obtained and

- their clinical impact.

This may be edited to ensure that readers understand the significance and context of the research. Please refer to any of our published articles for an example.

12) Author contributions: You will be asked to provide CRediT (Contributor Role Taxonomy) terms in the submission system. These replace a narrative author contribution section in the manuscript.

13) A Conflict of Interest statement should be provided in the main text.

14) Every published paper includes a 'Synopsis' to further enhance discoverability. Synopses are displayed on the journal webpage and are freely accessible to all readers. They include a short stand first (maximum of 300 characters, including space) as well as 2-5 one-sentences bullet points that summarizes the paper. Please write the bullet points to summarize the key NEW findings. They should be designed to be complementary to the abstract - i.e. not repeat the same text. We encourage inclusion of key acronyms and quantitative information (maximum of 30 words / bullet point). Please use the passive voice. Please attach these in a separate file or send them by email, we will incorporate them accordingly.

Please also suggest a striking image or visual abstract to illustrate your article as a PNG file 550 px wide x 300 px high.

15) Include a Reagents and Tools Table as part of the Methods section, which can be downloaded from our author guidelines.

EMBO Molecular Medicine has a "scooping protection" policy, whereby similar findings that are published by others during review or revision are not a criterion for rejection. Should you decide to submit a revised version, I do ask that you get in touch after three months if you have not completed it, to update us on the status.

*Additional important information regarding Figures

Each figure should be given in a separate file and should have the following resolution:
Graphs 800-1,200 DPI
Photos 400-800 DPI
Colour (only CMYK) 300-400 DPI"

Figures are not edited by the production team. All lettering should be the same size and style; figure panels should be indicated by capital letters (A, B, C etc). Gridlines are not allowed except for log plots. Figures should be numbered in the order of their appearance in the text with Arabic numerals. Each Figure must have a separate legend and a caption is needed for each panel.

*Additional important information regarding figures and illustrations can be found at
https://media.springernature.com/original/springer-cms/rest/v1/content/27825798/data/v1

***** Reviewer's comments *****

Referee #2 (Remarks for Author):

The authors have addressed my concerns.

Referee #3 (Remarks for Author):

The authors have sufficiently addressed all my questions.

The authors addressed the remaining editorial issues.

19th Jun 2026

Dear Prof. Lee,

We are pleased to inform you that your manuscript is accepted for publication and is now being sent to our publisher to be included in the next available issue of EMBO Molecular Medicine.

Your manuscript will be processed for publication by EMBO Press. It will be copy edited and you will receive page proofs prior to publication. Please note that you will be contacted by Springer Nature Author Services to complete licensing and payment information.

You may qualify for financial assistance for your publication charges - either via a Springer Nature fully open access agreement or an EMBO initiative. Check your eligibility: https://link.springer.com/journal/44321/how-to-publish-with-us

Should you be planning a Press Release on your article, please notify the production team via this form in order to coordinate publication and release dates: https://forms.cloud.microsoft/e/eH4A8JVP6g

If you have any questions, please do not hesitate to contact the Editorial Office. Thank you for your contribution to EMBO Molecular Medicine.

Yours sincerely,
Zeljko Durdevic

Zeljko Durdevic
Senior Editor
EMBO Molecular Medicine

>>> Please note that it is EMBO Molecular Medicine policy for the transcript of the editorial process (containing referee reports and your response letter) to be published as an online supplement to each paper. If you do NOT want this, you will need to inform the Editorial Office via email immediately. More information is available here: https://link.springer.com/partners/embo-press/editorial-policies#Peer%20review

EMBO Press Author Checklist

Corresponding Author Name: Yoontae Lee	
Journal Submitted to: EMBO Molecular Medicine	
Manuscript Number: EMM-2026-23286-V2	

USEFUL LINKS FOR COMPLETING THIS FORM
The EMBO Journal - Author Guidelines
EMBO Reports - Author Guidelines
Molecular Systems Biology - Author Guidelines
EMBO Molecular Medicine - Author Guidelines

Reporting Checklist for Life Science Articles (updated January
This checklist is adapted from Materials Design Analysis Reporting (MDAR) Checklist for Authors. MDAR establishes a minimum set of requirements in transparent reporting in the life sciences (see Statement of Task: 10.31222/osf.io/9sm4x). Please follow the journal's guidelines in preparing your
Please note that a copy of this checklist will be published alongside your article.

Abridged guidelines for figures
1. Data
The data shown in figures should satisfy the following conditions:
- the data were obtained and processed according to the field's best practice and are presented to reflect the results of the experiments in an accurate and unbiased manner.
- ideally, figure panels should include only measurements that are directly comparable to each other and obtained with the same assay.
- plots include clearly labeled error bars for independent experiments and sample sizes. Unless justified, error bars should not be shown for technical
- if n<5, the individual data points from each experiment should be plotted. Any statistical test employed should be justified.
- Source Data should be included to report the data underlying figures according to the guidelines set out in the authorship guidelines on Data

2. Captions
Each figure caption should contain the following information, for each panel where they are relevant:
- a specification of the experimental system investigated (eg cell line, species name).
- the assay(s) and method(s) used to carry out the reported observations and measurements.
- an explicit mention of the biological and chemical entity(ies) that are being measured.
- an explicit mention of the biological and chemical entity(ies) that are altered/varied/perturbed in a controlled manner.
- the exact sample size (n) for each experimental group/condition, given as a number, not a range;
- a description of the sample collection allowing the reader to understand whether the samples represent technical or biological replicates (including how many animals, litters, cultures, etc.).
- a statement of how many times the experiment shown was independently replicated in the laboratory.
- definitions of statistical methods and measures:
  - common tests, such as t-test (please specify whether paired vs. unpaired), simple χ2 tests, Wilcoxon and Mann-Whitney tests, can be unambiguously identified by name only, but more complex techniques should be described in the methods section;
  - are tests one-sided or two-sided?
  - are there adjustments for multiple comparisons?
  - exact statistical test results, e.g., P values = x but not P values < x;
  - definition of 'center values' as median or average;
  - definition of error bars as s.d. or s.e.m.

Please complete ALL of the questions below.
Select "Not Applicable" only when the requested information is not relevant for your study.

Materials

Newly Created Materials	Information included in the manuscript?	In which section is the information available? (Reagents and Tools Table, Materials and Methods, Figures, Data Availability Section)
New materials and reagents need to be available; do any restrictions apply?	Not Applicable	

Antibodies	Information included in the manuscript?	In which section is the information available? (Reagents and Tools Table, Materials and Methods, Figures, Data Availability Section)
For **antibodies** provide the following information:<br>- Commercial antibodies: RRID (if possible) or supplier name, catalogue number and or/clone number<br>- Non-commercial: RRID or citation	Yes	Reagents and Tools

DNA and RNA sequences	Information included in the manuscript?	In which section is the information available? (Reagents and Tools Table, Materials and Methods, Figures, Data Availability Section)
**Short novel DNA or RNA including primers, probes**: provide the sequences.	Yes	Reagents and Tools

Cell materials	Information included in the manuscript?	In which section is the information available? (Reagents and Tools Table, Materials and Methods, Figures, Data Availability Section)
**Cell lines:** Provide species information, strain. Provide accession number in repository **OR** supplier name, catalog number, clone number, and/**OR** RRID.	Not Applicable	
**Primary cultures:** Provide species, strain, sex of origin, genetic modification status.	Yes	Materials and Methods/ Reagents and Tools
Report if the cell lines were recently **authenticated** (e.g., by STR profiling) and tested for mycoplasma contamination.	Not Applicable	

Experimental animals	Information included in the manuscript?	In which section is the information available? (Reagents and Tools Table, Materials and Methods, Figures, Data Availability Section)
**Laboratory animals or Model organisms:** Provide species, strain, sex, age, genetic modification status. Provide accession number in repository **OR** supplier name, catalog number, clone number, **OR** RRID.	Yes	Materials and Methods
**Animal observed in or captured from the field:** Provide species, sex, and age where possible.	Yes	Materials and Methods
Please detail **housing and husbandry conditions**.	Yes	Materials and Methods

Plants and microbes	Information included in the manuscript?	In which section is the information available? (Reagents and Tools Table, Materials and Methods, Figures, Data Availability Section)
**Plants:** provide species and strain, ecotype and cultivar where relevant, unique accession number if available, and source (including location for collected wild specimens).	Not Applicable	
**Microbes:** provide species and strain, unique accession number if available, and source.	Not Applicable	

Human research participants	Information included in the manuscript?	In which section is the information available? (Reagents and Tools Table, Materials and Methods, Figures, Data Availability Section)
If collected and within the bounds of privacy constraints report on age, sex and gender or ethnicity for all study participants.	Yes	Materials and Methods

Core facilities	Information included in the manuscript?	In which section is the information available? (Reagents and Tools Table, Materials and Methods, Figures, Data Availability Section)
If your work benefited from core facilities, was their service mentioned in the acknowledgments section?	Yes	Materials and Methods

Design

Study protocol	Information included in the manuscript?	In which section is the information available? (Reagents and Tools Table, Materials and Methods, Figures, Data Availability Section)
If study protocol has been **pre-registered, provide DOI in the manuscript**. For clinical trials, provide the trial registration number **OR** cite DOI.	Not Applicable	
Report the **clinical trial registration number** (at ClinicalTrials.gov or equivalent), where applicable.	Not Applicable	

Laboratory protocol	Information included in the manuscript?	In which section is the information available? (Reagents and Tools Table, Materials and Methods, Figures, Data Availability Section)
Provide DOI OR other citation details if **external detailed step-by-step protocols** are available.	Yes	Materials and Methods

Experimental study design and statistics	Information included in the manuscript?	In which section is the information available? (Reagents and Tools Table, Materials and Methods, Figures, Data Availability Section)
Include a statement about **sample size** estimate even if no statistical methods were used.	Not Applicable	
Were any steps taken to minimize the effects of subjective bias when allocating animals/samples to treatment (e.g. **randomization procedure**)? If yes, have they been described?	Yes	Materials and Methods
Include a statement about **blinding** even if no blinding was done.	Not Applicable	
Describe **inclusion/exclusion criteria** if samples or animals were excluded from the analysis. Were the criteria pre-established?<br><br>If sample or data points were omitted from analysis, report if this was due to attrition or intentional exclusion and provide justification.	Not Applicable	
For every figure, are **statistical tests** justified as appropriate? Do the data meet the assumptions of the tests (e.g., normal distribution)? Describe any methods used to assess it. Is there an estimate of variation within each group of data? Is the variance similar between the groups that are being statistically compared?	Yes	Materials and Methods and Figures

Sample definition and in-laboratory replication	Information included in the manuscript?	In which section is the information available? (Reagents and Tools Table, Materials and Methods, Figures, Data Availability Section)
In the figure legends: state number of times the experiment was **replicated** in laboratory.	Yes	Figures
In the figure legends: define whether data describe **technical or biological replicates**.	Yes	Figures

Ethics

Ethics	Information included in the manuscript?	In which section is the information available? (Reagents and Tools Table, Materials and Methods, Figures, Data Availability Section)
Studies involving **human participants**: State details of **authority granting ethics approval** (IRB or equivalent committee(s), provide reference number for approval.	Yes	Materials and Methods
Studies involving **human participants**: Include a statement confirming that **informed consent** was obtained from all subjects and that the experiments conformed to the principles set out in the WMA Declaration of Helsinki and the Department of Health and Human Services Belmont Report.	Yes	Materials and Methods
Studies involving **human participants: For publication of patient photos**, include a statement confirming that consent to publish was obtained.	Not Applicable	
Studies involving experimental **animals**: State details of **authority granting ethics approval** (IRB or equivalent committee(s), provide reference number for approval. Include a statement of compliance with ethical regulations.	Yes	Materials and Methods
Studies involving **specimen and field samples:** State if relevant **permits** obtained, provide details of authority approving study; if none were required, explain why.	Not Applicable	

Dual Use Research of Concern (DURC)	Information included in the manuscript?	In which section is the information available? (Reagents and Tools Table, Materials and Methods, Figures, Data Availability Section)
Could your study fall under dual use research restrictions? Please check biosecurity documents and list of **select agents and toxins** (CDC): https://www.selectagents.gov/sat/list.htm	Not Applicable	
If you used a select agent, is the security level of the lab appropriate and reported in the manuscript?	Not Applicable	
If a study is subject to dual use research of concern regulations, is the name of the **authority granting approval and reference number** for the regulatory approval provided in the manuscript?	Not Applicable	

Reporting

The MDAR framework recommends adoption of discipline-specific guidelines, established and endorsed through community initiatives. Journals have their own policy about requiring specific guidelines and recommendations to complement MDAR.

Adherence to community standards	Information included in the manuscript?	In which section is the information available? (Reagents and Tools Table, Materials and Methods, Figures, Data Availability Section)
State if relevant guidelines or checklists (e.g., **ICMJE, MIBBI, ARRIVE, PRISMA**) have been followed or provided.	Not Applicable	
For **tumor marker prognostic studies,** we recommend that you follow the **REMARK** reporting guidelines (see link list at top right). See author guidelines, under 'Reporting Guidelines'. Please confirm you have followed these guidelines.	Not Applicable	
For **phase II and III randomized controlled trials**, please refer to the **CONSORT** flow diagram (see link list at top right) and submit the CONSORT checklist (see link list at top right) with your submission. See author guidelines, under 'Reporting Guidelines'. Please confirm you have submitted this list.	Not Applicable	

Data Availability

Data availability	Information included in the manuscript?	In which section is the information available? (Reagents and Tools Table, Materials and Methods, Figures, Data Availability Section)
Have **primary datasets** been deposited according to the journal's guidelines (see 'Data Deposition' section) and the respective accession numbers provided in the Data Availability Section?	Not Applicable	
Were **human clinical and genomic datasets** deposited in a public access-controlled repository in accordance to ethical obligations to the patients and to the applicable consent agreement?	Not Applicable	
Are **computational models** that are central and integral to a study available without restrictions in a machine-readable form? Were the relevant accession numbers or links provided?	Not Applicable	
If publicly available data were reused, provide the respective **data citations in the reference list**.	Not Applicable	
